# DiCARN-DNase: enhancing cell-to-cell Hi-C resolution using dilated cascading ResNet with self-attention and DNase-seq chromatin accessibility data

**DOI:** 10.1093/bioinformatics/btaf452

**Published:** 2025-08-13

**Authors:** Samuel Olowofila, Oluwatosin Oluwadare

**Affiliations:** Department of Computer Science, University of Colorado, Colorado Springs, CO, 80918, United States; Department of Computer Science, University of Colorado, Colorado Springs, CO, 80918, United States; Department of Biomedical Informatics, University of Colorado Anschutz Medical Campus, CO, 80045, United States; Department of Computer Science and Engineering, University of North Texas, Denton, TX, 76207, United States; Center for Computational Life Sciences, University of North Texas, Denton, TX, 76207, United States

## Abstract

**Motivation:**

The spatial organization of chromatin is fundamental to gene regulation and essential for proper cellular function. The Hi-C technique remains the leading method for unraveling 3D genome structures, but the limited availability of high-resolution (HR) Hi-C data poses significant challenges for comprehensive analysis. Deep learning models have been developed to predict HR Hi-C data from low-resolution counterparts. Early Convolutional Neural Network (CNN)-based models improved resolution but struggled with issues like blurring and capturing fine details. In contrast, Generative Adversarial Network (GAN)-based methods encountered difficulties in maintaining diversity and generalization. Additionally, most existing algorithms perform poorly in cross-cell line generalization, where a model trained on one cell type is used to enhance HR data in another cell type.

**Results:**

In this work, we propose Dilated Cascading Residual Network (DiCARN) to overcome these challenges and improve Hi-C data resolution. DiCARN leverages dilated convolutions and cascading residuals to capture a broader context while preserving fine-grained genomic interactions. Additionally, we incorporate DNase-seq data into our model, providing a robust framework that demonstrates superior generalizability across cell lines in HR Hi-C data reconstruction.

**Availability and implementation:**

DiCARN is publicly available at https://github.com/OluwadareLab/DiCARN

## 1 Introduction

Chromosome Conformation Capture (3C) technology is a molecular method used to analyze the spatial organization of chromatin in a cell (Lieberman-Aiden *et al.* 2009). The technology provides insights into the 3D architectural arrangement of chromosomes, allowing researchers to study the physical interactions between DNA segments that may be separated by large genomic distances along the linear genome. In recent genetics research, high-throughput chromosome conformation capture (Hi-C) has emerged as the preferred 3C technique for deciphering and analyzing spatial genome organization within the eukaryotic cell nuclei. It is a genome-wide approach to the study of 3D chromatin conformation inside the nucleus (Lieberman-Aiden *et al.* 2009). Lately, Hi-C has been the trailblazer technique in the exploration and characterization of genomic structural components, including A/B compartments, topologically associating domains (TADs) (Dixon *et al.* 2012), frequently interacting regions (Schmitt *et al.* 2016), stripes (Vian *et al.* 2018), and enhancer–promoter interactions (Rao *et al.* 2014). Being a biochemical approach that allows for an all-versus-all mapping of chromosomal and genome fragment interactions, Hi-C takes into account the interaction between pair-read assays generated from a wet lab process, resulting in a symmetric (*n* × *n*) contact matrix representation of the interaction frequencies (IFs), where *n* is the number of cells or evenly sized divisions of the genome called bins. The number indicated in every matrix cell represents the count of paired-end reads across 2 bins. The sizes of these bins, also known as “resolution,” habitually range from 1 kilobase (kb) to 2.5 megabase (Mb), whose range hinges on the sequencing depth. The relevance of Hi-C data is spiking geometrically owing to its practicability in elucidating the genome organization (Oluwadare *et al.* 2019).

However, a critical challenge in this research domain is the limited availability of the required Hi-C resolution for exhaustive studies of genomic structures. This challenge has inspired the use of deep learning (DL) models to predict the required high-resolution (HR) Hi-C data from the more readily available low-resolution (LR) variants, sharing interest similarities with the single-image super-resolution problem in the computer vision domain (He *et al.* 2016).

Zhang *et al.* (2018) pioneered HR Hi-C data prediction with HiCPlus, a Convolutional Neural Network (CNN)-based model inspired by SRCNN (Dong *et al.* 2014), which used a three-layer CNN to impute HR IFs. HiCNN (Liu and Wang 2019), a 54-layer CNN was modeled after DRRN (Tai *et al.* 2017). Both methods laid the groundwork for using CNNs in Hi-C enhancement, with subsequent models focusing on addressing challenges in improving resolution and generalization. SRHiC (Li and Dai 2020) introduced a ResNet-based approach (He *et al.* 2016) for Hi-C data enhancement, followed by the 2020 development of Generative Adversarial Network (GAN)-based models like DeepHiC (Hong *et al.* 2020) and HiCSR (Dimmick 2020), which improved resolution enhancement by utilizing generator-discriminator networks. Later, HiCARN (Hicks and Oluwadare 2022) introduced a more efficient cascading GAN, while DFHiC (Wang *et al.* 2023) advanced the field with a dilated full convolution network, preserving positional information and addressing previous shortcomings.

Despite these improvements, challenges such as limited receptive fields, lack of global context, and instabilities, particularly with mode collapse in GAN-based methods like HiCSR and DeepHiC, persist. Mode collapse results from the generator’s failure to produce diverse, representative samples, leading to incomplete data reconstruction. Additionally, most existing approaches have focused on architectural enhancements without integrating biologically relevant data, such as chromatin accessibility data, that reveals the chromosomal regions actively involved in gene regulation, which could provide more robust HR enhancement. In addition, existing algorithms perform poorly for cross-cell line generalization, where a model is trained on one cell and used for HR enhancement of another cell. This limitation significantly affects the model’s scalability and applicability in broader biological research, where variability across cell lines is common.

In this work, we propose Dilated Cascading Residual Network (DiCARN), a novel approach to overcoming these challenges. DiCARN improves model stability by employing dilated convolutions for a larger receptive field and incorporates chromatin accessibility data, enabling more accurate and biologically meaningful Hi-C resolution enhancement.

## 2 Materials and methods

### 2.1 Architecture

**Figure 1.**
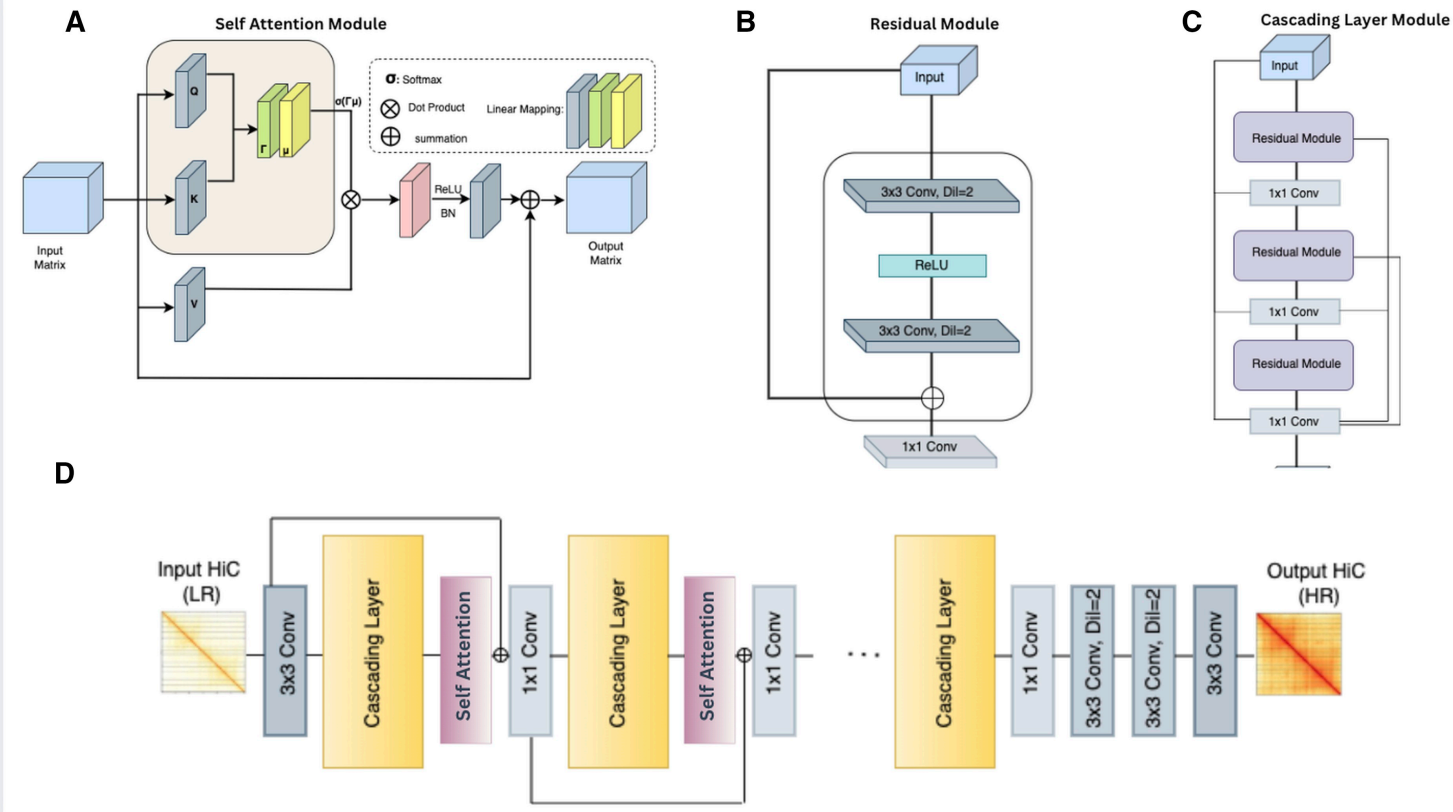
The DiCARN architecture comprises four major components. (A) Self-attention module that follows every instance of the cascading layer. (B) Residual module, which includes two dilated 3×3 convolutions, an ReLU activation function, a skip connection, and the 1×1 convolution in the concluding part. (C) Cascading layer, which encapsulates the residual modules separated by 1×1 convolutions. (D) Compressed visualization of the entire pipeline.

Our proposed model, DiCARN, implements a novel fusion of dilated convolutions (Shi *et al.* 2017), spatial self-attention (Vaswani *et al.* 2017, Zhang *et al.* 2022), and cascaded residual networks (Ahn *et al.* 2018), with its visual outlay depicted in Fig. 1.

#### 2.1.1 Cascading ResNet

Hicks and Oluwadare (2022) in their study performed a detailed ablation of the contributory impact of stacking Residual Networks (ResNets) (Ahn *et al.* 2018) in a cascade. Based on this study, DiCARN employs a serialized cascade (Fig. 1C) of multiple ResNet modules (Ahn *et al.* 2018). Each residual module consists of two 3×3 convolutional layers, followed by a ReLU activation function, and incorporates a skip connection, enabling the network to retain information from earlier layers. This architectural design enhances the training process by mitigating issues such as vanishing gradients. Furthermore, feature representation is progressively refined in each cascading layer, as outputs from successive residual modules are combined to enhance the network’s overall performance. We posit that adopting the cascading ResNet in our architecture will mitigate the vanishing gradient problem.

#### 2.1.2 Dilation

Dilated convolution has been widely recognized for its ability to expand the receptive field without increasing the number of parameters, allowing for efficient integration of long-range dependencies in spatial data (Lin *et al.* 2018), a concept on which Hi-C data enhancement pivots. Prior studies have demonstrated that dilation provides an exponential increase in the field of view while maintaining computational efficiency, making it particularly useful for tasks that require capturing fine-grained structural information (Shi *et al.* 2017). Unlike pooling operations, which reduce spatial resolution, dilated convolutions preserve the feature map size, ensuring that crucial spatial details remain intact (Wang *et al.* 2019). Typically, the kernels in a convolution are contiguous. Dilation follows the à trous algorithm, a technique used to increase the receptive field of the convolution operation by spacing out the kernel points without incrementing the number of parameters or the filter size (Zhang *et al.* 2015). “Trous” is a French term for “with holes,” essentially describing the implementation of dilated convolutions as the inclusion of gaps in the vanilla convolution operation.


(1) $$\varphi_{i}=\sum_{1}^{k}x[i+d\times k]\times w[k]$$

Equation (1) expresses this concept mathematically, where $\varphi_{i}$ is the computed feature map, *d* is the dilation rate, *k* denotes kernel size, *w*[*k*] symbolizes the kernel weights, and *x*[*i*] signifies the input feature map. We use dilated convolution within the residual module (Fig. 1B) of our cascading layers and at the tail end of the entire network (Fig. 1D) just before the final enhanced output is produced.

Since dilated convolutions expand the receptive field without increasing computational cost, enabling the model to capture both local and distal genomic interactions in Hi-C data. We posit that adopting this component will enhance the model’s ability to reconstruct HR structures by effectively incorporating information across multiple genomic scales. In Section 3.1, we present ablation experiments to evaluate the effect of the dilation step in our Hi-C data enhancement model.

#### 2.1.3 Spatial self-attention

One of the long-standing challenges contending with CNN-based methods is their mode of treating all data point (loci) equally, thereby fostering redundancy in their computation of LR features (Lu and Hu 2022, Zhang *et al.* 2022). In their super-resolution work, Zeng *et al.* (2023) adopted spatial self-attention due to its ability to suppress noise while dynamically adjusting feature importance in super-resolution tasks. Similarly, Chen *et al.* (2021) proposed an “Attention in Attention Network” for image super-resolution, which integrates attention mechanisms to enhance feature representation. Given these precedents, we posit that employing such attention-based methods could have a comparable effect in Hi-C resolution enhancement.

In this work, we adopt the spatial self-attention method from Vaswani *et al.* (2017), incorporating it into our approach (Fig. 1A) to dynamically focus on different regions of the original LR feature map. This mechanism enhances the model’s ability to capture complex spatial dependencies and vital contextual information across spatial dimensions. In Section 3.1, we present ablation experiments to evaluate the effect of the spatial attention module on our Hi-C data enhancement model.

### 2.2 Loss function

The DiCARN model training employs the mean squared error (MSE) loss function, leveraging its effectiveness in minimizing the difference between predicted and target values. This choice also aims to ensure computational simplicity and an exclusive focus on error minimization between predicted and observed Hi-C matrices. Equation (2) depicts the MSE, where *m* is the IF dimension, $P_{a,b}$ and $Q_{a,b}$ are the ground truth and predicted IFs between distal loci *a* and *b*, respectively.


(2) $$\text{MSE}=\frac{1}{m^{2}}\sum_{a,b}{\left(P_{a,b}-Q_{a,b}\right)}^{2}$$

### 2.3 Data and preprocessing

Our choice of Hi-C dataset is informed by the work of Rao *et al.* (2014), which provides Hi-C data for several human cell types (K562, HMEC, NHEK, and GM12878). All datasets are available in the NCBI GEO Accession Database under Accession ID: GSE63525. We also used human Foreskin Fibroblast-1 (HFF-hTERT) cell line, from the 4DN database repository (ID: 4DNESU36SE4V) (Akgol Oksuz *et al.* 2021). We trained our model using the GM12878 cell data, ensuring balance by excluding the X and Y chromosomes to avoid sex-related biases. Following the random chromosome selection method used by Hicks and Oluwadare (2022), validation was performed using chromosomes 2, 6, 10, and 12, while chromosomes 1–22, excluding chromosomes 4, 14, 16, and 20, were used for training. These excluded chromosomes were later utilized across cell lines for testing our model. For usability ease, all data used was split into small blocks of 40×40 dimensions.

### 2.4 Evaluation metrics

To ensure a fair comparison, we adopt the structural similarity index measure (SSIM) and the peak signal-to-noise ratio (PSNR), the two favored computational metrics used in this research domain (Hong *et al.* 2020). We also used GenomeDISCO (Ursu *et al.* 2018) for our concordance measure and HiCRep (Yang *et al.* 2017) for the assessment of biological reproducibility.


(3) $$\mathrm{SSIM}(x,y)=\frac{\left(2\mu_{x}\mu_{y}+C_{1})(2\sigma_{xy}+C_{2}\right)}{\left(\mu_{x}^{2}+\mu_{y}^{2}+C_{1})(\sigma_{x}^{2}+\sigma_{y}^{2}+C_{2}\right)}$$

The SSIM between two images *x* and *y* is mathematically expressed as shown in Equation (3) where $\mu_{x}$ and $\mu_{y}$ are the mean intensities of images *x* and *y*, $\sigma_{x}^{2}$ and $\sigma_{y}^{2}$ are the variances of images *x* and *y*, $\sigma_{xy}$ is the covariance of images *x* and *y*, $C_{1}$ and $C_{2}$ are the stability constants.

Equation (4) gives the mathematical representation of the PSNR between two images.


(4) $$\mathrm{PSNR}=10\cdot {\;\text{log}\;}_{10}\left(\frac{L^{2}}{\mathrm{MSE}}\right)$$

*L* is the maximum possible pixel value of the image (e.g. 255 for 8-bit images), while MSE is the mean squared error between the given images.

Equation (5) shows the mathematical derivation of the concordance score as proffered by GenomeDISCO.


(5) $$S(A_{1},A_{2})=1-D(A_{1,t},A_{2,t})$$

The concordance score given by this formula ranges between −1 and 1, where higher values signify greater similarity between the subject contact maps. Given two denoised contact maps $A_{1,t}$ and $A_{2,t}$, the difference between them is calculated using the distance $L_{1}$ as shown in Equation (6).


(6) $$D(A_{1,t},A_{2,t})=\frac{1}{N}\sum_{i,j}\left|A_{1,t}(i,j)-A_{2,t}(i,j)\right|$$

HiCRep, whose computation is presented in Equation (7) is the measure of the stratum-adjusted correlation coefficient (SCC) where $X_{k}$ and $Y_{k}$ are the contact frequencies contained in the stratum *k*, $\text{cov}(X_{k},Y_{k})$ is the measure of covariance between $X_{k}$ and $Y_{k}$, $\text{var}(X_{k})$ and $\text{var}(Y_{k})$ are the variances of $X_{k}$ and $Y_{k}$ within every stratum, *K* is the sum total of strata.


(7) $$\text{SCC}=\frac{\sum_{k=1}^{K}\mathrm{cov}(X_{k},Y_{k})}{\sqrt{\sum_{k=1}^{K}\mathrm{var}(X_{k})\sum_{k=1}^{K}\mathrm{var}(Y_{k})}}$$

## 3 Results

### 3.1 Hyperparameter search

Our proposed model is hinged on three key hyperparameters. (i) number of cascading layers: HiCARN (Hicks and Oluwadare 2022) performed a detailed hyperparameter search to determine the optimal number of cascading blocks in their work. They found that five cascading blocks provided the optimal result. Hence, we adopted the same number of blocks. (ii) Dilation rate: to determine the dilation rate, we performed a hyperparameter search across dilation rates 2–5 and configurations. Our results show that a dilation rate of 2, with a configuration involving two dilated convolutions in the residual block as featured in Fig. 1B and a dilated convolution stack at the end of the network (Fig. 1D), produced the optimal result (Tables S1 and S2, available as supplementary data at *Bioinformatics* online). We performed an ablation study to assess the impact of the spatial attention mechanism on the DiCARN model with and without it. The ablation study shows that DiCARN has superior performance with the incorporation of the dilation component (Table S4, available as supplementary data at *Bioinformatics* online). (iii) Self-attention: to determine how to incorporate self-attention, we experimented with different configurations and the application of self-attention in different layers of our cascade architecture. Our optimal model was obtained by applying spatial self-attention to only the first two cascade blocks (Fig. 1D), as shown in Table S3, available as supplementary data at *Bioinformatics* online. We also perform an ablation study of the impact of incorporating the self-attention component, the result shows a superior performance with the incorporation of the self-attention in DiCARN (Table S5, available as supplementary data at *Bioinformatics* online).

### 3.2 Training, validation, and testing

**Figure 2.**
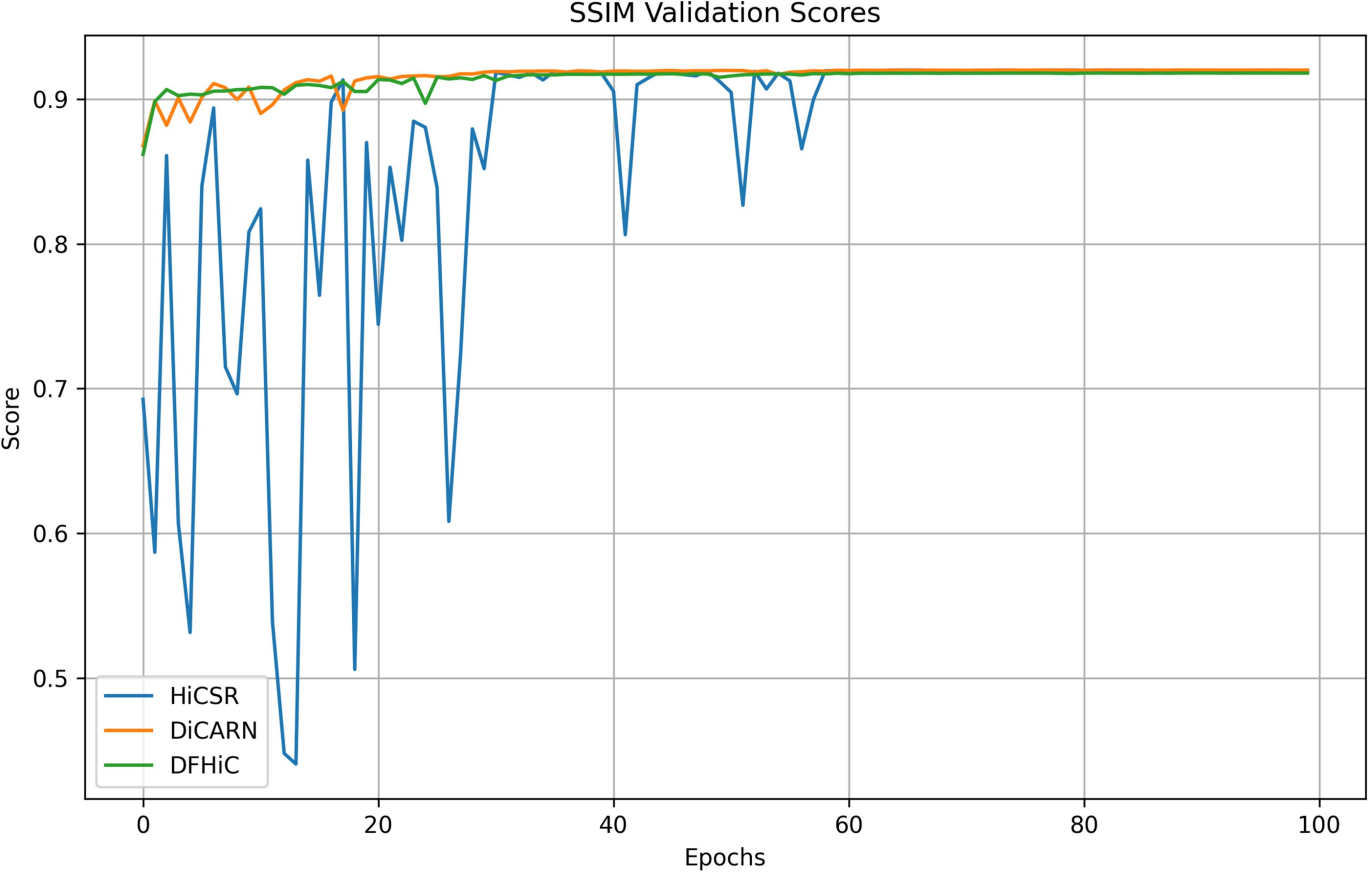
Validation result for DiCARN and state-of-the-art algorithms. Using the SSIM metric, the DiCARN validation results are contrasted with two state-of-the-art methods, HiCSR and DFHiC.

In the training phase, we conduct a validation after every training epoch so that the progressive performance of the model is accurately tracked and the optimally performing model weights are saved accordingly. This validation performance is then benchmarked against existing state-of-the-art methods (Fig. 2). More results are presented in Fig. S1, available as supplementary data at *Bioinformatics* online. The trainings were done using the LR Hi-C dataset downsampled from the 10 kb HR variant made available in the GEO database accession number GSE63525. All models were trained on an NVIDIA GeForce RTX 4090 GPU with 24 GB of VRAM, and the system had 128 GB of RAM.

### 3.3 DiCARN performance on same cell line data

HiCSR (Dimmick 2020) and DFHiC (Wang *et al.* 2023) were selected for comparison with our model due to their demonstrated efficacy in GAN-based and CNN-based Hi-C data enhancement pipelines, respectively. We also selected DeepHiC (Hong *et al.* 2020) and iEnhance (Li *et al.* 2023) due to their use of composite loss functions and their demonstrated superior performance in Hi-C data enhancement. After training our model on the 40 kb LR GM12878 Hi-C dataset, DiCARN consistently outperformed state-of-the-art models in both computational efficiency and biological benchmarks when tested on previously unseen GM12878 chromosomes. As shown in Table 1, DiCARN’s same-cell prediction results exhibit superior performance relative to existing models. Additionally, we evaluated the model’s performance using a 1/64 downsample ratio, with the corresponding results provided in Table S6, available as supplementary data at *Bioinformatics* online. Table 2 includes results to assess generalization to unseen and diverse datasets. In this work, we kept Table 1 focused on the same-cell setting (i.e. train on GM12878 cell line and test on GM12878 cell line) to preserve clarity and make the results easier to interpret, while Table 2 represents results for unseen and diverse cell types (i.e. train on GM12878 cell line and test on other cell lines). As shown in the tables, the mean and standard deviation of the evaluation metrics demonstrate that DiCARN consistently produces stable results, often exhibiting lower or comparable standard deviations relative to other methods. The training time and peak memory usage of the examined models are documented in Figs S2 and S3, available as supplementary data at *Bioinformatics* online, respectively.

**Table 1.** DiCARN is benchmarked against existing methods based on same-cell GM12878 data with a downsampling ratio of 1/16.

Metric	Method	Chr4	Chr14	Chr16	Chr20	Mean ± SD
**SSIM**	HiCSR	0.9300	0.9101	0.9080	0.9086	0.9142 ± 0.0106
	DFHiC	0.9292	0.9093	0.9055	0.9074	0.9128 ± 0.0110
	DeepHiC	0.9200	0.9017	0.8963	0.9001	0.9045 ± 0.0106
	iEnhance	0.8769	0.8500	0.8258	0.8465	0.8498 ± 0.0210
	DiCARN	0.9315	0.9120	0.9111	0.9097	**0.9161** ± 0.0103
**PSNR**	HiCSR	36.8908	35.4460	33.9085	34.9226	35.2920 ± 1.2424
	DFHiC	36.7807	35.3461	33.6090	34.7944	35.1326 ± 1.3163
	DeepHiC	36.6777	35.2864	33.5139	34.7479	35.0565 ± 1.3110
	iEnhance	32.8958	30.7501	28.7821	29.5899	30.5045 ± 1.7872
	DiCARN	36.9576	35.4686	33.9062	34.9067	**35.3098** ± 1.2745
**GenomeDISCO**	HiCSR	0.9146	0.9219	0.9040	0.9256	0.9165 ± 0.0095
	DFHiC	0.9151	0.9220	0.9045	0.9253	0.9167 ± 0.0092
	DeepHiC	0.9041	0.9129	0.8991	0.9167	0.9082 ± 0.0080
	iEnhance	0.9100	0.9163	0.8951	0.9188	0.9101 ± 0.0106
	DiCARN	0.9175	0.9243	0.9073	0.9273	**0.9191** ± 0.0089
**HiCRep**	HiCSR	0.9179	0.8932	0.9408	0.8621	0.9035 ± 0.0338
	DFHiC	0.9178	0.8928	0.9406	0.8620	0.9033 ± 0.0338
	DeepHiC	0.9164	0.8918	0.9398	0.8602	0.9021 ± 0.0341
	iEnhance	0.8898	0.8806	0.9289	0.8473	0.8867 ± 0.0336
	DiCARN	0.9188	0.8928	0.9409	0.8622	**0.9037** ± 0.0339

The mean ± standard deviation (SD) column reports the average metric value across the test chromosomes. The result values in bold fonts signify the best scores.

**Table 2.** Performance benchmarking of state-of-the-art methods in contrast with DiCARN across unseen and diverse cell lines using average scores on test data.

Cell line	Method	SSIM	PSNR	MSE	HiCRep (Mean ± SD)
K562 (Rao *et al.* 2014)	HiCSR	0.9485	34.7512	**0.0003**	0.8414 ± 0.0424
	DFHiC	0.9472	34.1669	**0.0003**	0.8773 ± 0.044
	DiCARN	**0.9498**	**35.2087**	**0.0003**	**0.8807** ± 0.0424
	DeepHiC	0.9357	35.398	0.0003	0.8728 ± 0.0450
	iEnhance	0.8913	32.1213	0.0006	0.8540 ± 0.0436
HMEC (Rao *et al.* 2014)	HiCSR	0.9748	35.2839	**0.0002**	0.7572 ± 0.0532
	DFHiC	0.9737	34.7582	0.0003	0.7578 ± 0.0529
	DiCARN	**0.9757**	**35.2237**	**0.0002**	**0.765** ± 0.0477
	DeepHiC	0.9582	35.1826	**0.0002**	0.762 ± 0.0506
	iEnhance	0.9147	31.8546	0.0006	0.7246 ± 0.0519
NHEK (Rao *et al.* 2014])	HiCSR	0.9721	35.093	**0.0002**	**0.8014** ± 0.0539
	DFHiC	0.9705	34.0983	0.0003	0.802 ± 0.0544
	DiCARN	**0.9739**	**35.4481**	**0.0002**	0.8007 ± 0.0549
	DeepHiC	0.9567	35.1298	**0.0002**	0.7947 ± 0.0509
	iEnhance	0.9119	31.8316	0.0007	0.7947 ± 0.0509
HFF- hTERT (Akgol Oksuz *et al.* 2021)	HiCSR	0.9777	42.6452	**0.0001**	0.6235 ± 0.0631
	DFHiC	0.9783	42.8118	**0.0001**	0.6211 ± 0.0620
	DiCARN	**0.9802**	**42.9884**	**0.0001**	**0.6256** ± 0.0621
	DeepHiC	0.9564	42.0052	**0.0001**	0.5843 ± 0.0631
	iEnhance	0.9239	41.0953	**0.0001**	0.5337 ± 0.0734

The HiCRep column reports the mean ± standard deviation (SD) across the test chromosomes. The result values in bold font signify the best scores. The results suggest that DiCARN retains its ability to restore the fidelity of *in silico* Hi-C data from unseen cell lines.

### 3.4 DiCARN generalizability test across unseen cell lines

**Figure 3.**
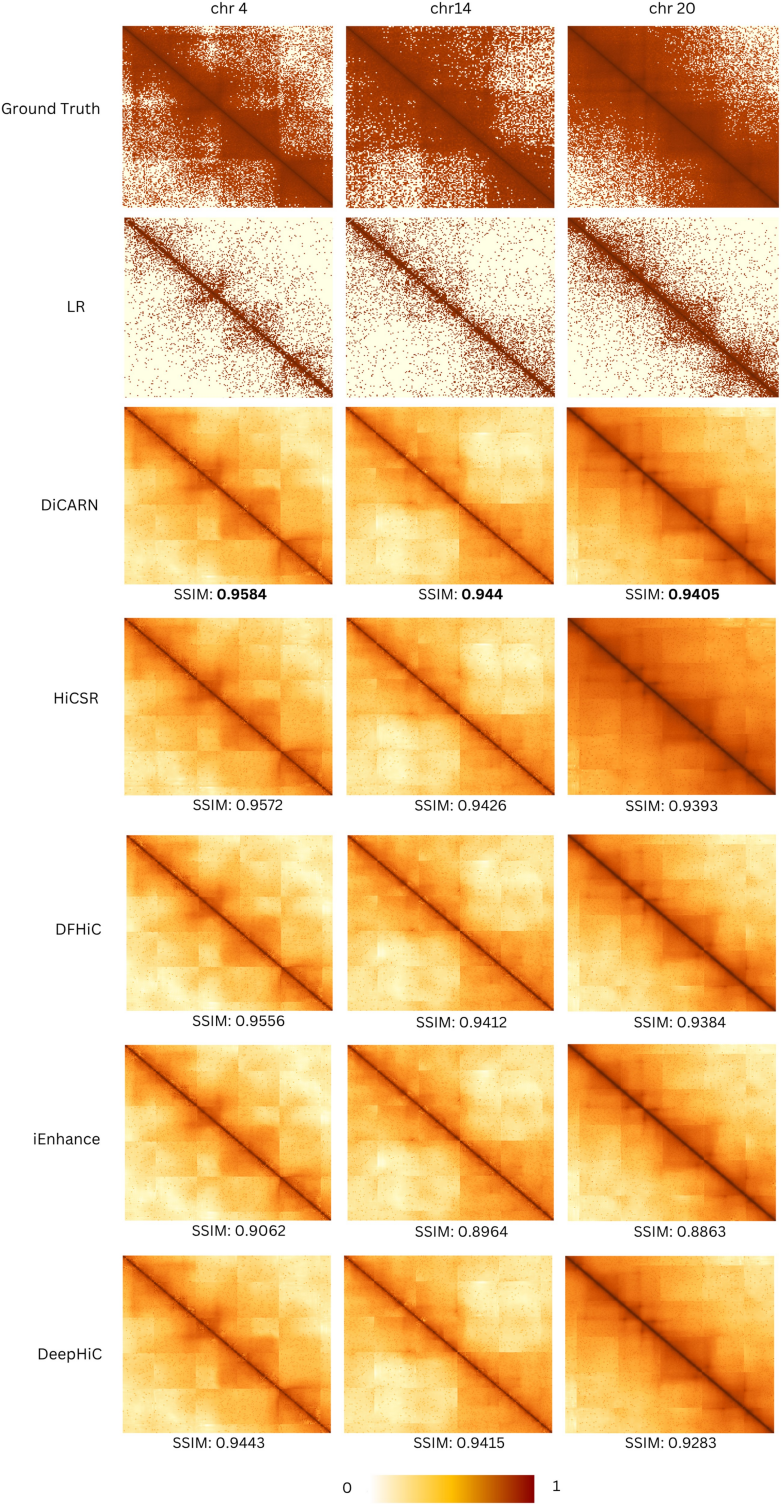
Heatmap visualization of the cross-cell enhancement results for DiCARN in comparison with state-of-the-art models (HiCSR, DFHiC, iEnhance, and DeepHiC) on chromosomes 4, 14, and 20 of the K562 cell line.

Having trained the models on GM12878 cell type data only, we show in Table 2 that DiCARN generalizes better than existing state-of-the-art methods on the lymphoblast cell (K562), the mammary epithelial cell (HMEC), the human epidermal keratinocytes cell (NHEK), and the human foreskin fibroblast-1 cell (HFF-hTERT) (Akgol Oksuz *et al.* 2021). The experiment in this phase is based on datasets downsampled at a 1/16 ratio for both training and testing. The results show that DiCARN consistently demonstrates superior generalization to unseen cell lines across all datasets based on computational metrics, and in all but one cell line based on biological metrics—HiCRep (Table 2) and GenomeDISCO (Table S7, available as supplementary data at *Bioinformatics* online). In Table 2, the HiCRep column reports the mean and standard deviation across the test chromosomes, demonstrating the stability of our model’s performance across different cell lines. We also present a visualization comparison of the corresponding structure similarity index measure for chromosomes 4, 14, and 20 for the different algorithms in Fig. 3.

### 3.5 DiCARN accurately enhances chromatin loop features

**Figure 4.**
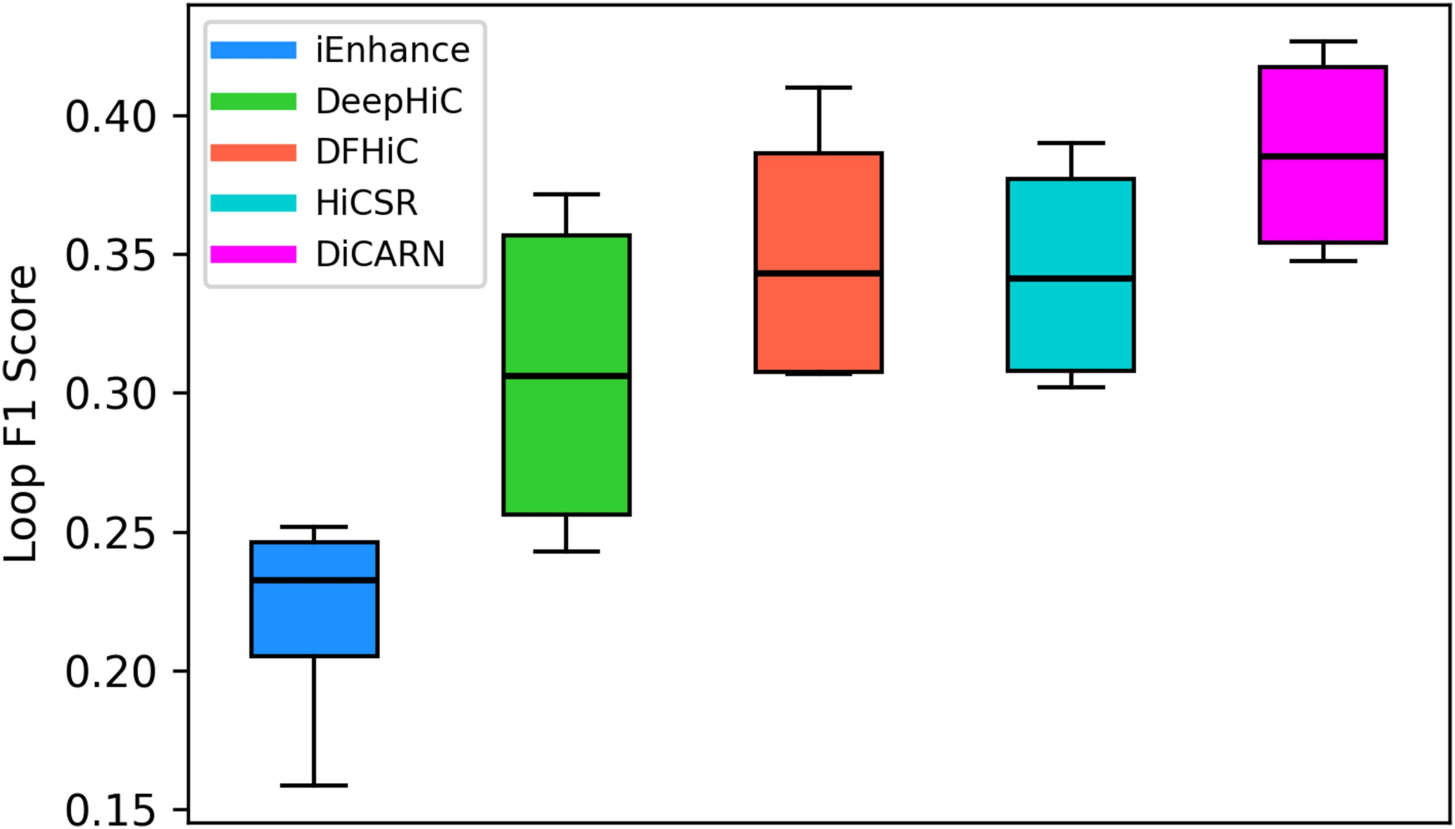
Model performance comparison on cross-cell datasets using loop analysis. Using loop F1 score analysis, we evaluated loops detected from predicted K562 cell line matrices generated by models trained on the GM12878 cell line.

We used Mustache (Ardakany *et al.* 2020), a peak-calling algorithm for Hi-C data, to evaluate the enhancement capabilities of different models in reconstructing chromatin interactions. We quantified performance using the loop F1 score, comparing predicted loops against the ground truth to assess how well each model recovers biologically relevant chromatin interactions. The loop F1 scores across K562 cell line chromosomes 4, 14, 16, and 20 indicate that DiCARN consistently outperforms other models, as presented in Fig. 4 and Table S8, available as supplementary data at *Bioinformatics* online. DiCARN consistently achieves higher loop F1 scores across these four chromosomes, indicating an enhanced capability to detect biologically meaningful interactions.

### 3.6 Enhancing generalizability with chromatin accessibility data from DNase-seq

In this study, we used DNase-seq epigenomic data, building on prior findings from Tan *et al.* (2023), Yang *et al.* (2023), Wang *et al.* (2022). Yang *et al.* (2023) demonstrated that different epigenomic tracks data, including DNase-seq and histone modifications, can compensate for each other in Hi-C contact prediction, suggesting redundancy. Similarly, studies by Yang *et al.* (2023) and Tan *et al.* (2023) show that DNase-seq with CTCF ChIP-seq and ATAC-seq with CTCF ChIP-seq, respectively, perform comparably for Hi-C contact map prediction, further supporting redundancy among epigenomic signals. Meanwhile, Wang *et al.* (2022) incorporated DNase-seq into 3D genome reconstruction, showing that DNase-seq improves the accuracy of inferred 3D structures and enables cell-type-specific Hi-C contact prediction. Collectively, these studies establish DNase-seq as a highly effective independent predictor of Hi-C interactions. Given its broad coverage, predictive strength, and demonstrated utility in contact map prediction and 3D genome reconstruction, we adopt DNase-seq as the primary chromatin accessibility signal in DiCARN-DNase for Hi-C data enhancement. Based on this, we propose a novel approach for HR Hi-C enhancement that leverages DNase-seq data to address the limitations of conventional Hi-C enhancement algorithms. We derive IFs from the DNase-seq and ultimately utilize these data to augment our training set and improve the generalizability of our model. The IF derived from DNase-seq is cell-type specific and is expected to enable accurate predictions across different biological contexts.

To calculate asynchronous IFs from DNase-seq, we employ a linear regression model, as shown in Equation (8) (Wang *et al.* 2022).

(8) $$\epsilon_{k,l}=\alpha_{1}R_{k}+\alpha_{2}R_{l}+\alpha_{3}D_{k,l}$$

Our DNase-based IF imputation procedure, which enhances the resolution of 3D genomic maps, begins with the normalization of raw Hi-C interaction counts using Knight–Ruiz (KR) normalization to produce a normalized IF matrix. This matrix is then symmetrized to maintain consistency, and a pairwise distance matrix is generated to reflect spatial proximities among genomic loci. Due to the size of the interaction matrices, we fragment the data into manageable chunks, mapping each chunk to the corresponding DNase-seq signal using *bedtools* (Quinlan and Hall 2010), thereby aligning chromatin accessibility data with the genomic coordinates. The DNase signal across each fragment is averaged to provide a summary measure of chromatin accessibility for each genomic region. Using these genomic distances and DNase signals as input, we predict distances using the pretrained model defined in Equation (8) where $\epsilon_{k,l}$ represents the predicted IF between fragments *k* and *l*, $R_{k}$ and $R_{l}$ denote the DNase-seq signal levels for fragments *k* and *l*, $D_{k,l}$ is the 1D genomic distance between these fragments, while $\alpha_{1}$, $\alpha_{2}$, and $\alpha_{3}$ are fitting parameters derived from imputed 3D distances (Wang *et al.* 2022). Subsequently, the imputed distances are converted into IFs (Lieberman-Aiden *et al.* 2009). The final reassembly process involves combining these IF matrix fragments into a complete matrix for the chromosome, followed by a KR normalization to ensure consistency with the original Hi-C data. Ultimately, this process produces a DNase-inferred IF matrix that supplements Hi-C data to refine resolution and improve interpretability across diverse cell types.

#### 3.6.1 Improving generalizability across unseen cell lines with DNase-seq data

To further enhance the generalizability of our model, we incorporate DNase-inferred IF data into the existing training dataset through a targeted data augmentation strategy intended to bolster the model’s predictive capacity across various cell lines. This strategy is explored in two configurations. In the target DNase scenario, DNase-imputed IF data from the target cell line’s test chromosomes is appended to the original Hi-C training dataset, after which the augmented dataset is used to retrain the DiCARN model. This model is called DiCARN-DNase-T (see illustration in Fig. S4, available as supplementary data at *Bioinformatics* online). Specifically, these chromosomes correspond to those downsampled for testing. *We hypothesize that DNase-seq inferred IF data from the target cell type provides valuable insights into Hi-C interactions, facilitating enhanced model generalizability across varying biological contexts*.

The source DNase scenario, on the other hand, utilizes DNase-inferred IF data from the source cell type’s training chromosomes and adds it to the source dataset for training. This model is called DiCARN-DNase-S (see illustration in Fig. S5, available as supplementary data at *Bioinformatics* online). *We hypothesize that including source DNase-based data not only augments the training set but also endows the model with a deeper understanding of chromatin dynamics beyond the source cell type.*

In this study, the source dataset is the GM12878 Hi-C dataset, and the target cell is the cell line on which we are testing the generalization.

Given that DNase data is expected to improve biological reproducibility, we evaluate our model’s performance using Hi-C analysis metrics the SCC through HiCRep, and the concordance score by GenomeDISCO. These metrics provide more biologically significant analysis measures compared to standard image evaluation metrics.

### 3.7 DiCARN-DNase: enhancing DiCARN with DNase-seq for cross-cell line generalization

Table 3 highlights the HiCRep scores obtained for both configurations (DiCARN-DNase-T and DiCARN-DNase-S), where we demonstrate that at least one implementation of the DNase-augmented models outperforms the vanilla DiCARN model. The GenomeDISCO scores are presented in Table S9 and Fig. S6, available as supplementary data at *Bioinformatics* online.

**Table 3.** HiCRep average score comparison of DiCARN and its DNase-based variants on 1/16 downsampled datasets from multiple cell lines.

Cell	Method	Chr4	Chr14	Chr16	Chr20	Mean ± SD
K562	DiCARN	0.893	0.8466	0.9354	0.8478	0.8807 ± 0.0380
	DiCARN-DNase-S	0.8914	0.8452	0.9334	0.8469	0.8792 ± 0.0381 ↓
	DiCARN-DNase-T	0.8931	0.8466	0.9417	0.8465	**0.884 **± 0.0408 ↑
HMEC	DiCARN	0.7302	0.7794	0.8193	0.6986	0.7570 ± 0.0496
	DiCARN-DNase-S	0.7181	0.7716	0.8140	0.6923	0.7490 ± 0.0508 ↓
	DiCARN-DNase-T	0.7223	0.7752	0.8187	0.7132	**0.7574 **± 0.0465 ↑
NHEK	DiCARN	0.8161	0.8158	0.8493	0.7217	0.8007 ± 0.0541
	DiCARN-DNase-S	0.8172	0.8153	0.8482	0.7293	**0.8012 **± 0.0504 ↑
	DiCARN-DNase-T	0.8195	0.8196	0.8516	0.7312	**0.8055 **± 0.0502 ↑

The last column reports Mean ± SD across the four chromosomes. ↑ implies better result than vanilla DiCARN. ↓ implies worse result than vanilla DiCARN, while the result values in bold fonts signify the best scores.

**Figure 5.**
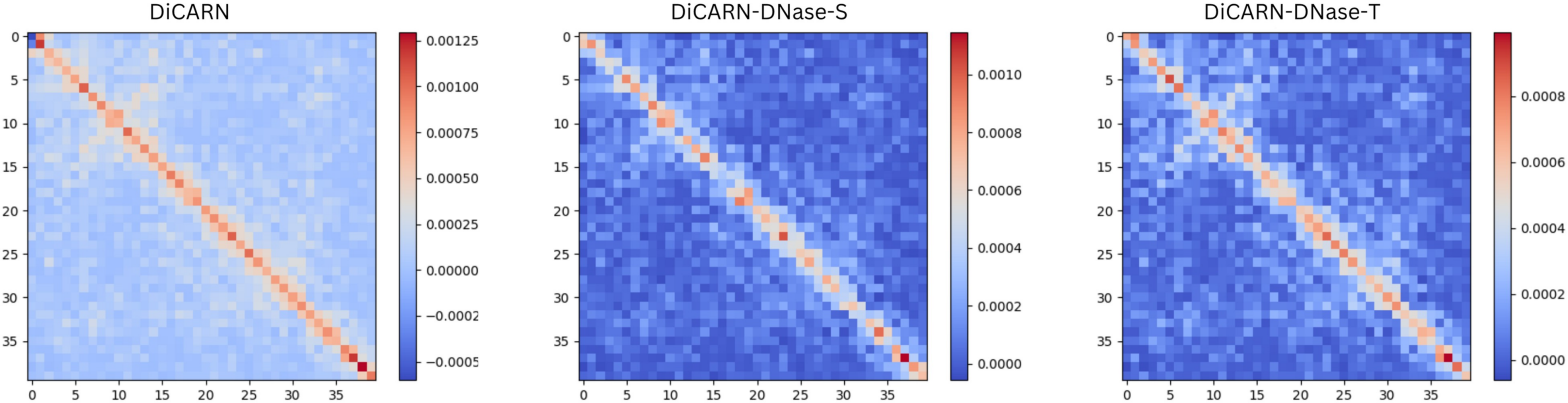
Heatmap visualization of the SHAP feature enrichment analysis for the three DiCARN variants. The figure shows the 40×40 heatmap of the high SHAP-valued regions predicted by the DiCARN-DNase-Target model (right), along with the corresponding 40×40 region from the predicted contact matrix for DiCARN (left) and DiCARN-DNase-Source (middle) models. The DiCARN-DNase-Target model captures richer interaction patterns, particularly along the diagonal.

To explain the performance of our super-resolution models, particularly those incorporating DNase-inferred IF data, we employed SHapley Additive exPlanations (SHAP) (Lundberg and Lee 2017) values to interpret the contribution of different regions within the predicted 40×40 Hi-C matrices; a negative SHAP value indicates that a feature or region decreases the predicted interaction relative to the model’s baseline output, while a positive value indicates a feature or region increases it. Specifically, we aggregated the SHAP values across each matrix and generated boxplots to compare the distribution of feature importance scores between models. Models that demonstrated more stable and higher SHAP value distributions in key regions suggest they are more effectively capturing critical genomic interaction patterns. This finding aligns with their improved performance in both computational and biological reproducibility metrics. The boxplots of the SHAP values (Fig. S7, available as supplementary data at *Bioinformatics* online) show that the DNase-Target variant consistently outperforms the others. Table 4 presents the rankings of the SHAP analysis, which further support our hypothesis that the DNase-Target model captures more biologically relevant regions, making its predictions more similar to the ground truth. In contrast, incorporating DNase-Source data resulted in worse performance compared to the vanilla (no DNase) model in two of the three cases (Table 3), suggesting that the DNase-Source data introduces inconsistencies, which likely stem from cell-type specific differences and biological variability between the source DNase-seq data and the target Hi-C data. These inconsistencies negatively impact the model’s performance. The predominance of negative SHAP values (Table 4; Fig. S7, available as supplementary data at *Bioinformatics* online), particularly when averaged across samples, suggests that many input features contribute less or negatively impact the predicted HR contact values, especially bins that are farther off from the diagonal. Notably, among the models presented, DNase-Target model shows the fewest negatively contributing features, as reflected by its comparatively higher (though still negative) mean SHAP value in Table 4. This suggests that DNase-Target’s features, on average, contribute more positively or less suppressively to the prediction, which may underlie its improved performance. Figure 5 shows the 40×40 heatmap of the high SHAP-valued regions predicted by the DNase-Target model, along with the corresponding 40×40 region from the predicted contact matrix. As an example for this 40×40 region, the DiCARN model heatmap (left) includes more features with negative SHAP values at corresponding positions compared to the other models, indicating that these features contribute less or negatively impact the final prediction for this region. The results demonstrate that the DNase-Target model captures richer interaction patterns, particularly along the diagonal, which suggests improved modeling of proximal genomic interactions compared to other models. Also, reporting lower standard deviation values in most of the cases, suggesting a higher level of stability in the results (Table 3). In addition, other tests performed on computational metrics—such as training time, and GPU memory usage—demonstrated that the DNase-Target variant is superior in terms of efficiency (Table 4). Consequently, we conclude that DNase-Source data are too inconsistent to provide a reliable improvement in our framework.

**Table 4.** Comparison of SHAP statistics, training time, and GPU overhead across different DiCARN models.

Model	SHAP, Mean ± SD	SHAP, Median	Training time (min)	GPU (MB)
DiCARN	−0.024615 ± 0.031745	−0.035329	123	6114
DiCARN-DNase-S	−0.024819 ± 0.032019	−0.035612	339	8120
DiCARN-DNase-T	−**0.023977 **± 0.030750	−**0.034380**	178	6200

The best result is presented in bold.

Ultimately, since the DNase-Target hypothesis resulted in the most consistent improvements across biological and computational metrics, we designate the DNase-Target variant as the definitive DiCARN-DNase model. Additionally, Section S2.2, available as supplementary data at *Bioinformatics* online, describes an evaluation of the impact of DNase-Inferred IF data on Hi-C resolution enhancement, demonstrating that DNase-inferred IF contributes meaningful structural information, even when high-quality Hi-C data are available (Fig. S7, available as supplementary data at *Bioinformatics* online).

### 3.8 Enhancing generalizability of state-of-the-art models with DNase-seq data

Following the enhancement capability boost recorded by DiCARN when influenced by the IF imputed from the DNase-seq data—that is, augmented with the Target DNase variant now designated as the default DNase, we proceeded to appropriate these data augmentation innovation to some existing state-of-the-art (SOTA) models in the Hi-C resolution enhancement research domain, including HiCSR (Dimmick 2020), HiCARN (Hicks and Oluwadare 2022), HiCNN (Liu and Wang 2019), DFHiC (Wang *et al.* 2023), DeepHiC (Hong *et al.* 2020), and iEnhance (Li *et al.* 2023) to test the generalizability of the DNase idea to other models on K562, HMEC, and NHEK at 1/16 ratio. From the HiCRep results presented in Table 5, it is observed that the data augmentation approach worked in 10 of 12 scenarios. More results are provided in Table S10 and Fig. S8, available as supplementary data at *Bioinformatics* online. It is also observed that the DFHiC was the base method in the two instances where the approach was challenged. However, the majority of results obtained from the test for applicability to other methods established the proposition that the fusion of DNase with *in silico* LR GM12878 Hi-C data improves the cell-to-cell Hi-C reproducibility capabilities of deep learning-based methods. This DFHiC exception leads us to believe that the proposed data augmentation approach might be sensitive to the algorithm of the method.

**Table 5.** Using HiCRep for reproducibility assessment, we show the performance scores of the DNase-based data augmentation approach for six existing SOTA models across three cell lines at 1/16 downsampling ratio.

Cell	Method	Chr4	Chr14	Chr16	Chr20	**Mean** ± **SD**
K562	HiCSR	0.8913	0.8444	0.9304	0.8414	0.8769 ± 0.0383
	HiCSR-DNase	0.8923	0.8445	0.9304	0.8441	**0.8778** ± 0.0379
	DFHiC	0.8905	0.8423	0.9338	0.8424	**0.8773** ± 0.0398
	DFHiC-DNase	0.8869	0.8398	0.9307	0.8367	0.8735 ± 0.0399
	HiCARN	0.8928	0.8444	0.9375	0.8496	**0.8811** ± 0.0394
	HiCARN-DNase	0.8913	0.8424	0.9324	0.849	0.8788 ± 0.0384
	HiCNN	0.8956	0.8477	0.9257	0.8378	0.8767 ± 0.0358
	HiCNN-DNase	0.8903	0.8434	0.9359	0.847	**0.8792** ± 0.0376
	DeepHiC	0.8867	0.8352	0.9306	0.8386	0.8728 ± 0.0407
	DeepHiC-DNase	0.8941	0.8483	0.9308	0.8425	**0.8789** ± 0.0372
	iEnhance	0.8674	0.8314	0.9074	0.8097	0.8540 ± 0.0401
	iEnhance-DNase	0.8687	0.8413	0.9074	0.8078	**0.8563** ± 0.0365
HMEC	HiCSR	0.7296	0.7793	0.8201	0.6998	0.7572 ± 0.0494
	HiCSR-DNase	0.74	0.7734	0.8554	0.694	**0.7657** ± 0.0637
	DFHiC	0.7328	0.7775	0.8212	0.6997	0.7578 ± 0.0494
	DFHiC-DNase	0.738	0.7844	0.8244	0.7019	**0.7622** ± 0.0506
	HiCARN	0.7269	0.776	0.8191	0.6947	0.7542 ± 0.0504
	HiCARN-DNase	0.7312	0.7796	0.8218	0.7012	**0.7585** ± 0.0498
	HiCNN	0.7182	0.7711	0.8144	0.6952	0.7497 ± 0.0464
	HiCNN-DNase	0.7257	0.7762	0.8163	0.6957	**0.7535** ± 0.0463
	DeepHiC	0.7386	0.7854	0.8221	0.7020	0.7620 ± 0.0501
	DeepHiC-DNase	0.7383	0.7804	0.824	0.7112	**0.7635** ± 0.0428
	iEnhance	0.6784	0.7400	0.7954	0.6847	0.7246 ± 0.0538
	iEnhance-DNase	0.6892	0.7439	0.8032	0.681	**0.7293** ± 0.0490
NHEK	HiCSR	0.8165	0.8152	0.8498	0.7242	0.8014 ± 0.0519
	HiCSR-DNase	0.8194	0.817	0.8509	0.7283	**0.8039** ± 0.0510
	DFHiC	0.8168	0.8167	0.8504	0.7239	**0.8029** ± 0.0522
	DFHiC-DNase	0.8131	0.8152	0.8487	0.7177	0.7987 ± 0.0539
	HiCARN	0.8157	0.8136	0.8462	0.7229	0.7996 ± 0.0520
	HiCARN-DNase	0.8186	0.8163	0.8494	0.7266	**0.8027** ± 0.0514
	HiCNN	0.82	0.8159	0.8	0.73	0.7915 ± 0.0363
	HiCNN-DNase	0.8089	0.806	0.8392	0.717	**0.7928** ± 0.0456
	DeepHiC	0.8051	0.8066	0.8438	0.7232	0.7947 ± 0.0526
	DeepHiC-DNase	0.8218	0.8175	0.8511	0.732	**0.8056** ± 0.0444
	iEnhance	0.8051	0.8066	0.8438	0.7232	0.7946 ± 0.0526
	iEnhance-DNase	0.8096	0.7979	0.8282	0.7634	**0.7998** ± 0.0236

Each vanilla method is contrasted with its corresponding DNase variant (e.g. HiCSR-DNase). We observe that the majority of the test cases support the DNase proposition. The better result is in bold.

### 3.9 Benchmarking DiCARN-DNase against other methods across cell lines

The integration of DNase-seq data significantly enhances the performance of both our model and existing algorithms. To assess the overall performance across methods, we selected the better-performing variant of each algorithm—either the vanilla or the DNase-augmented version—per cell line and constructed a ranking table based on HiCRep scores, which measure reproducibility across three cell lines using the average from four test chromosomes. The scores for DiCARN are presented in Table 3, while the results for the other four algorithms are provided in Table 5. As shown in the ranking table (Table S11, available as supplementary data at *Bioinformatics* online), DiCARN demonstrated strong performance in two out of three cell lines—ranking first in K562 and second in NHEK—and remained competitive in HMEC. While no single method consistently outperformed others across all cell lines, these results highlight DiCARN’s potential in diverse biological contexts.

### 3.10 Benchmark on 3D genome reconstruction and TAD detection

**Figure 6.**
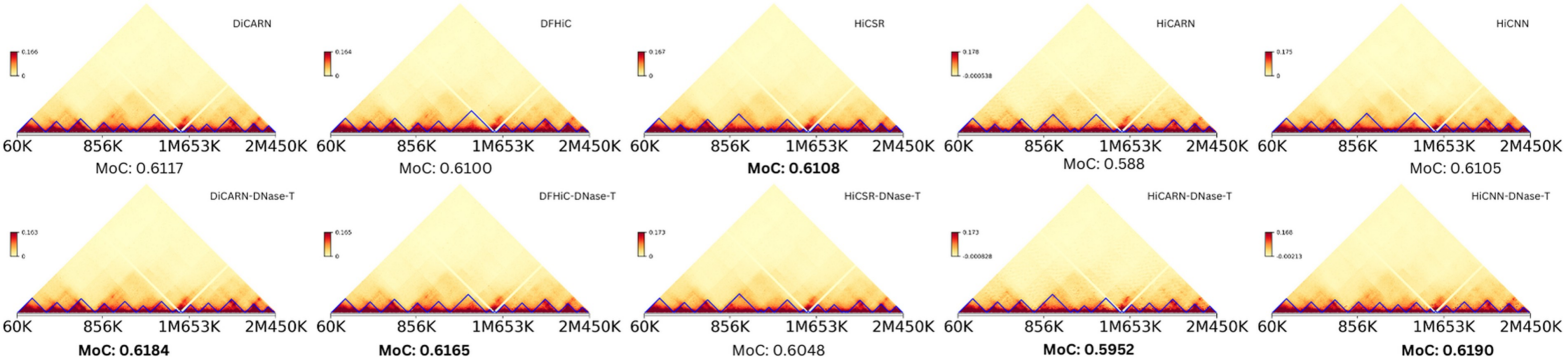
The TAD recovery assessment of the Baseline algorithm (vanilla) compared versus the DNase-based variants. We used the 60 kb–2.45 Mb region of the chr14 in the K562 cell line for this procedure. The procedure was also executed across other methods to show their TAD recovery abilities. The heatmaps are tagged with their corresponding MoCs to show the improvement of the DNase-based models on the vanilla variants.

The ability of the data from these models to recover TADs plays a critical role in exploring functional genomics and regulating gene expression by controlling enhancer–promoter interactions (Dixon *et al.* 2012) and also plays an important role toward usefulness. In this study, we employed TopDom (Shin *et al.* 2016) to detect TADs from region 60 kb to 2.45 Mb region of K562 cell line chromosome 14 using the imputed Hi-C data and the ground truth data. We assessed their concordance through the measure of concordance (MoC) metric (Higgins *et al.* 2022). A higher MoC score is better. The results indicate that the DNase-based variants for most algorithms closely match the ground truth, underscoring the impact of DNase-seq data on enhancing Hi-C data (Fig. 6).

**Figure 7.**
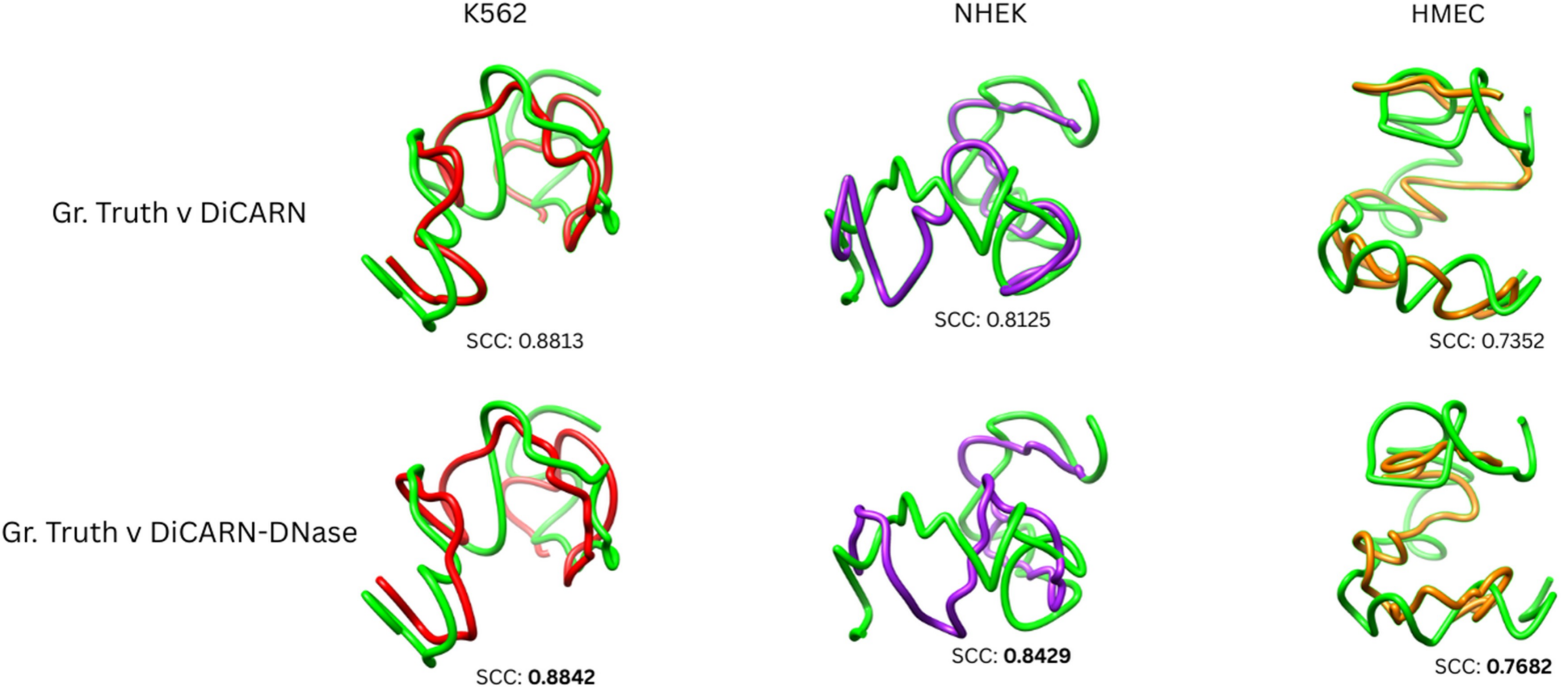
Evaluation of Groundtruth consistency with output from DiCARN variants for chromosome 20 of the K562 cell line using the 300–350 MB region. Reconstructed 3D structures for DNase-based DiCARN records more consistency based on the SCC scores than its vanilla variant.

Furthermore, we evaluated the structural similarity of the 3D genome reconstructed from both the imputed and ground truth data. Using 3DMax (Oluwadare *et al.* 2018), we reconstructed structures for region 300–350 Mb of K562 cell line chromosome 20 and compared them via the Spearman correlation coefficient (Fig. 7). The results demonstrate that the DNase-augmented DiCARN model showed greater concordance with the ground truth than the vanilla DiCARN model. Overall, these findings affirm the potency of DNase-seq augmentation in the prediction accuracy of 3D genomic structures.

### 3.11 Incorporating DNase impacts the discovery of unique biological chromatin loops

**Figure 8.**
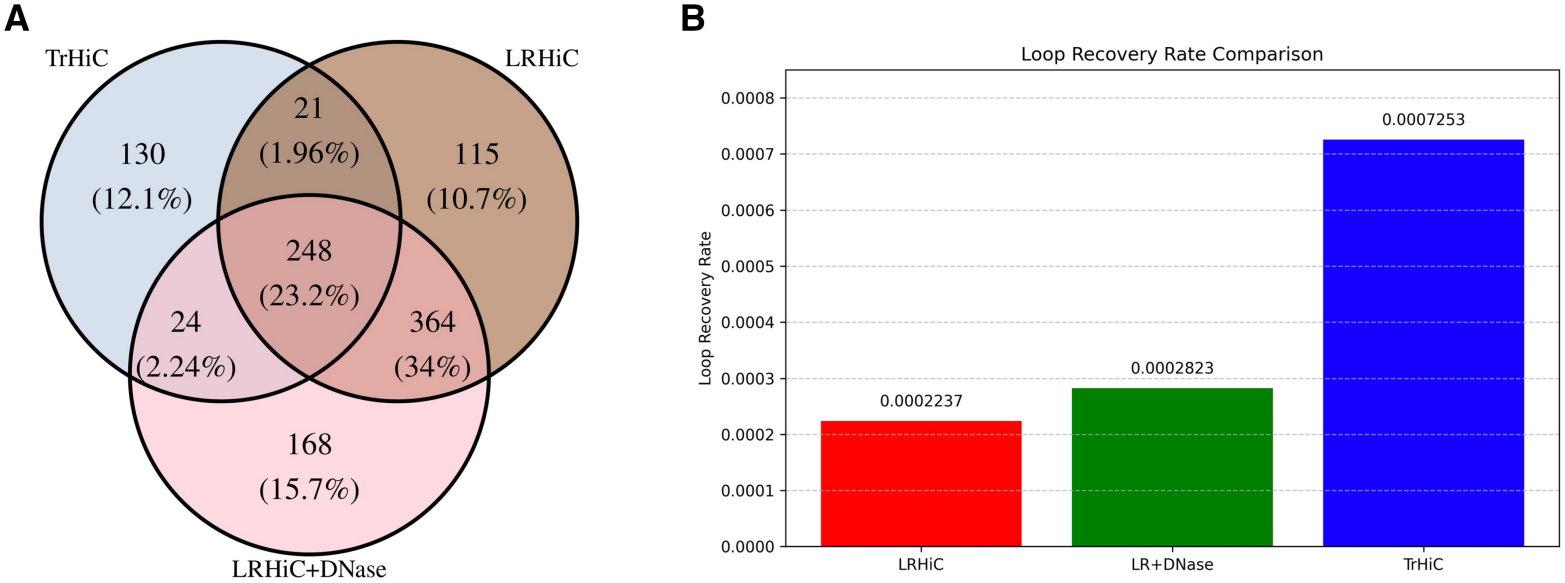
DNAse impact on biological chromatin loop recovery. (A) Venn diagram showing detected loop overlaps among TrHiC, LRHiC, and LRHiC+DNase models on chr20. DNase-enhanced model recovers more unique and shared loops than LRHiC. (B) Bar plots showing CTCF loop recovery rate for TrHiC, LRHiC, and LRHiC+DNase models on chr20.

To investigate whether the incorporation of chromatin accessibility data enables the enhanced Hi-C matrix to capture structural patterns not present in either LR or true HR Hi-C data (Fig. S9, available as supplementary data at *Bioinformatics* online), we conducted a loop detection analysis on chromosome 20 of the K562 cell line. We used Mustache (Ardakany *et al.* 2020), a robust peak-calling algorithm for loop detection from Hi-C data, to identify chromatin loops from three datasets: low-resolution Hi-C data (LrHi-C), DiCARN-DNase-enhanced Hi-C data (LrHi-C + DNase-inferred IF), and true HR Hi-C data (TrHi-C). We then used publicly available scripts from Mohit Chowdhury *et al.* (2025) to generate a Venn diagram showing the overlap of detected loops among the three cases. This allowed us to identify and assess the unique loops detected only in the DiCARN-DNase–enhanced Hi-C data, as well as those shared with either or both of the other datasets. Figure 8A shows that 23.2% of the loops are consistent across all datasets, with a strong overlap observed between LrHi-C and LrHi-C+DNase. Notably, LrHi-C+DNase and TrHi-C exhibit a higher overlap than LrHi-C and TrHi-C, suggesting that incorporating DNase data improves contact resolution for downstream analyses such as loop detection, compared to using LrHi-C alone. To evaluate the biological validity of the detected loops, we assessed CTCF enrichment at loop anchors across the different input conditions (Liu et al. 2022). Among all datasets (Fig. 8B), TrHi-C loops showed the highest overall CTCF recovery, indicating strong alignment with known regulatory protein binding. Interestingly, the unique loops identified by LrHi-C+DNase—those not detected by either LrHi-C or TrHi-C individually—achieved the second-highest CTCF recovery rate. This enrichment suggests that these additional loops are not false positives but rather represent biologically meaningful chromatin interactions. In contrast, loops uniquely identified by LrHi-C alone exhibited lower CTCF recovery, suggesting weaker regulatory support and potentially lower confidence.

## 4 Conclusion

In this study, we introduce DiCARN, an attention-based Dilated Cascading ResNet model for the recovery of HR Hi-C data necessary for biological and computational exploits of genomic structures. Eminently, our study pivots on the introduction of a novel approach involving distal inferences from the chromatin accessibility DNase data of human cell lines for the augmentation of LR IF data. The practicality of this innovation was tested and established using biological reproducibility and structural similarity metrics. It is important to note that the inclusion of DNase-seq data has been universally beneficial across all models, including existing state-of-the-art models. This study emphasizes how the use of DNase-seq data has elevated the performance of both our model and others.

## Supplementary Material

btaf452_Supplementary_Data

## Data Availability

DiCARN-DNase is a containerized software made available via: https://github.com/OluwadareLab/DiCARN_DNase. The DNase-seq data were collected from Roadmap and Consortia Database (https://egg2.wustl.edu/roadmap/web_portal/processed_data.html). The Hi-C datasets used in this study including GM12878, K562, HMEC, and NHEK (Rao *et al.* 2014) GEO Accession Database via GEO code GSE63525, while the HFF-hTERT (Akgol Oksuz *et al.* 2021) cell line is available in 4DN Dataset Portal with Title ID 4DNESU36SE4V. The CTCF data were obtained from the GEO Accession Database with ID GSM5454703 (https://www.ncbi.nlm.nih.gov/geo/query/acc.cgi? acc=GSM5454703) (Liu *et al.* 2022).
