# Supplementary material for: DiCARN-DNase: enhancing cell-to-cell Hi-C resolution using dilated cascading ResNet with self-attention and DNase-seq chromatin accessibility data
Source: Bioinformatics. 2025 Aug 13;41(9):btaf452. doi: 10.1093/bioinformatics/btaf452 (PMC12448792; doi:10.1093/bioinformatics/btaf452)
Supplement: btaf452_Supplementary_Data [file btaf452_supplementary_data.pdf]

SUPPLEMENTARY MATERIAL

DiCARN-DNase: Enhancing Cell-to-Cell Hi-C Resolution Using Dilated Cascading ResNet with Self-Attention and DNase-seq Chromatin Accessibility Data

Samuel Olowofila[1] and Oluwatosin Oluwadare[1,2,3,4,*]

[1]Department of Computer Science, University of Colorado at Colorado Springs, 1420 Austin Bluffs Pkwy, Colorado Springs, 80918, Colorado, USA, [2]Department of Biomedical Informatics, University of Colorado Anschutz Medical Campus, 13001 East 17th Place, Aurora, 80045, Colorado, USA, [3]Department of Computer Science and Engineering, University of North Texas, 3940 N. Elm St., Denton, 76207, TX, USA and [4]Center for Computational Life Sciences, University of North Texas, 76207, TX, USA
*Corresponding author. oluwatosin.oluwadare@unt.edu

1.

The DiCARN Architecture

1.1 Spatial Self Attention

Suppose that Hi-C feature map $B_i \subset \mathbb{R}^{HxWxD}$ (where i = q, k, v) is obtained post-convolutions from the spatial attention module input $B \subset \mathbb{R}^{HxWxD}$, denoting Query, Key, and Value respectively, we represent spatial attention operation as follows [1]:

$$\mathbf{B}_i = \mathbf{G}_i(\mathbf{B}), \quad i = q, k, v \tag{1}$$

where $C(.)$ represents the convolution, which results in a C-dimensional feature map matrix B_i, a feature map featuring C channels. B_i is then reshaped into a 2D form, expressed thus:

$$\mathbf{B}_{\text{reshape},i} = \mathbf{H}(\mathbf{B}_i), \quad i = q, k, v \tag{2}$$

After symmetrizing the query, key, and value outputs using the reshape function H, we follow up with a transpose and multiplication computation expressed as follows:

$$(\mathbf{B}_{\text{reshape},q})^T = \mathbf{U}(\mathbf{B}_{\text{reshape},q}) \tag{3}$$

Here, the U represents the transpose mapping. This operation is then followed by the computation of the weight coefficient matrix M expressed thus:

$$\mathbf{M} = (\mathbf{B}_{\text{reshape},1})^T \times \mathbf{B}_{\text{reshape},2} \tag{4}$$

Softmax is now applied to the obtained weight coefficient matrix M so that $M_{softmax}$ is then obtained:

$$b_{ij} = \frac{\exp(y_{ij})}{\sum_{k=1,l=1}^{N} \exp(y_{ij})}, \quad \mathbf{M}_{\text{softmax}} = [b_{ij}] \tag{5}$$

Finally, the attention feature map is computed as follows:

$$\mathbf{F} = \mathbf{B} + \beta \cdot \mathbf{U}(\mathbf{U}(\mathbf{M}_{\text{softmax}}) \times \mathbf{B}_{\text{reshape},3}) \tag{6}$$

1.2Hyperparameter Search

Table 3 reports our search for the optimal application of spatial self-attention in our architectural pipeline. The results reported informed our decision to fit the DiCARN model with spatial self-attention on the first 2 cascades.

The decision on the number of dilations that work optimally for our model was determined by a series of tests. The results shown in Table 1 informed our decision to use the dilation rate of 2 in every instance of dilated convolution within the architecture of our model.

As shown in Table 2, our decision to append two 3x3 convolutions with a dilation rate of 2 is informed by the results recorded in this phase of our ablation study. Based on the performance observations at varying instances of dilation application on the tail of our model architecture, we adopt a dual-layer convolution with a dilation rate of 2 in our final structure.

2. Results

2.1 DiCARN Same Cell Results on GM12878 1/64 ratio Downsampled Data

To further establish our hypothesis about the performance of DiCARN, we repeat the high-resolution data prediction experiment, this time training on 1/64 downsampled Hi-C data of all but four GM12878, which were used for testing. This result is presented in Table 6.

Table 1. Performance metrics at various dilation rates within the residual blocks of the DiCARN model network.

CHR	SSIM	PSNR	GenomeDISCO	Loss
Dilation Rate = 2				
3	0.9532	34.1225	0.8478	0.0004
11	0.9472	34.3523	0.8564	0.0003
19	0.9508	35.8801	0.8567	0.0003
21	0.9327	34.008	0.8677	0.0004
Average	0.946	34.5907	**0.8572**	**0.0003**
Dilation Rate = 3				
3	0.954	34.2952	0.8484	0.0004
11	0.9481	34.7978	0.8564	0.0003
19	0.951	36.1661	0.8564	0.0003
21	0.9333	34.4736	0.8669	0.0004
Average	**0.9466**	34.4736	0.857	**0.0003**
Dilation Rate = 4				
3	0.9538	34.464	0.8474	0.0004
11	0.9477	34.8228	0.8559	0.0003
19	0.9508	36.1773	0.8554	0.0003
21	0.9327	34.3966	0.8676	0.0004
Average	0.9463	**34.9652**	0.8567	**0.0003**
Dilation Rate = 5				
3	0.9372	34.4214	0.8193	0.0004
11	0.9304	34.1406	0.8283	0.0004
19	0.9331	33.4715	0.8283	0.0005
21	0.9116	33.4666	0.8393	0.0006
Average	0.9281	33.875	0.8288	0.0005

Table 2. Performance Metrics for DiCARN Models with varying dilated convolutional layers at the tail end of the architecture. *DiCARN + (2 X DL - Tail)* means the tail of the network comprises a stack of 2 dilated convolutions, etc.

CHR	SSIM	PSNR	GenomeDISCO
DiCARN + (1 x DL-Tail)			
3	0.9532	34.1225	0.8478
11	0.9472	34.3523	0.8564
19	0.9508	35.8801	0.8567
21	0.9327	34.008	0.8677
Average	0.946	34.5907	0.8572
DiCARN + (2 x DL-Tail)			
3	0.9535	34.4711	0.8473
11	0.9479	34.8474	0.8555
19	0.9514	36.152	0.8558
21	0.9337	34.4538	0.866
Average	0.9466	34.9811	0.856
DiCARN + (3 x DL-Tail)			
3	0.9536	34.5774	0.8396
11	0.9478	34.7324	0.8515
19	0.9513	35.7523	0.8516
21	0.9333	34.3046	0.8627
Average	0.9465	34.841675	0.8514
DiCARN + (5 x DL-Tail)			
3	0.9527	34.181	0.8448
11	0.9472	34.5606	0.855
19	0.9511	35.7801	0.8549
21	0.9329	34.1898	0.8667
Average	0.946	34.6779	0.8554

2.2. Evaluating the Impact of DNase-Inferred Interaction Frequency Data on Hi-C Resolution Enhancement

To assess the true impact of DNase-inferred interaction frequencies (IF) on Hi-C resolution enhancement, we performed a new comparison using True HR Hi-C (GroundTruth) + DNase-inferred IF for the K562 cell line. The results are presented in the supplementary material (Supplementary Figure S9). As expected, performance improved across conditions in the following order, from lowest to best: DiCARN without DNase (i.e., LrHi-C alone), LrHi-C + DNase-inferred IF, and finally True HR Hi-C +

Table 3. Average Scores of Attention on DiCARN Cascades

**Attention on DiCARN Cascades**			
**Average Scores**	**SSIM**	**PSNR**	**GenomeDISCO**
Att. 1 Cascade	0.9468	35.2252	0.8558
Att. 2 Cascades	0.9471	**35.2986**	**0.8579**
Att. 3 Cascades	0.9459	34.4689	0.8266
Att. 4 Cascades	**0.9474**	35.1339	0.8563
Att. 5 Cascades	**0.9474**	35.1815	0.8561

Table 4. Ablation Study: Performance Comparison of DiCARN Variants

**CHR**	**SSIM**	**PSNR**	**GenomeDISCO**
**DiCARN ->CARN w/o Dilation**			
3	0.9521	33.3308	0.8418
11	0.9468	33.8879	0.8515
19	0.9511	35.7365	0.8516
21	0.9328	33.7490	0.8656
**Average**	0.9457	34.1761	0.8526
**DiCARN ->CARN + Dilation**			
3	0.9532	34.1225	0.8478
11	0.9472	34.3523	0.8564
19	0.9508	35.8801	0.8567
21	0.9327	34.0080	0.8677
**Average**	0.9460	34.5907	0.8572

Table 5. Ablation Study: Further performance comparison of DiCARN Variants

**CHR**	**SSIM**	**PSNR**	**GenomeDISCO**
**{DiCARN + Dilation} w/o Attention**			
3	0.9532	34.1225	0.8478
11	0.9472	34.3523	0.8564
19	0.9508	35.8801	0.8567
21	0.9327	34.0080	0.8677
**Average**	0.9460	34.5907	0.8572
**{DiCARN + Dilation} + Attention**			
4	0.9544	35.0344	0.8491
14	0.9485	35.2864	0.8571
16	0.9514	36.0213	0.8571
20	0.9471	34.8432	0.8668
**Average**	**0.9471**	**35.2986**	**0.8579**

DNase-inferred IF, which achieved the highest result. These findings demonstrate that DNase-inferred IF contributes meaningful structural information, enhancing resolution even when high-quality Hi-C data is available.

Table 6. Comparison of same-cell SSIM, PSNR, GenomeDISCO, and HiCRep scores across four different chromosomes, using 1/64 downsampled GM12878 data. The last row for each method reports the Mean ± SD (standard deviation) across the four chromosomes. Best performing Mean ± SD (per metric) is in bold.

Method	Chromosome	SSIM	PSNR	GenomeDISCO	HiCRep
**HiCSR**	Chr 4	0.9143	35.6552	0.8985	0.6758
	Chr 14	0.8908	34.1203	0.9056	0.7477
	Chr 16	0.8709	31.6647	0.8797	0.8497
	Chr 20	0.8898	33.4203	0.9081	0.6697
	**Mean ± SD**	0.8915 ± 0.0178	**33.7151** ± 1.6552	0.8980 ± 0.0128	0.7357 ± 0.0838
**DFHiC**	Chr 4	0.9106	35.2824	0.8932	0.6746
	Chr 14	0.8873	33.8337	0.9010	0.7458
	Chr 16	0.8640	31.0145	0.8753	0.8491
	Chr 20	0.8854	33.0785	0.9038	0.6690
	**Mean ± SD**	0.8868 ± 0.0190	33.3023 ± 1.7783	0.8933 ± 0.0128	0.7346 ± 0.0839
**DiCARN**	Chr 4	0.9148	35.5979	0.8983	0.6783
	Chr 14	0.8909	34.1000	0.9055	0.7483
	Chr 16	0.8710	31.5100	0.8795	0.8504
	Chr 20	0.8897	33.3620	0.9100	0.6695
	**Mean ± SD**	**0.8916** ± 0.0180	33.6425 ± 1.6989	**0.8983** ± 0.0134	**0.7366** ± 0.0836

Table 7. Average + Standard Deviation GenomeDISCO scores for different models across various cell lines at 1/16 downsampling ratio.

**Cell Line**	**Models**	**GenomeDISCO** (Avg + SD)
K562	HiCSR	0.8287 ± 0.0199
	DFHiC	0.8343 ± 0.0191
	DiCARN	**0.8579** ± 0.0081
	DeepHiC	0.8189 ± 0.0199
	iEnhance	0.8227 ± 0.0214
HMEC	HiCSR	0.5111 ± 0.0832
	DFHiC	0.5239 ± 0.0841
	DiCARN	**0.5181** ± 0.0863
	DeepHiC	0.503 ± 0.086
	iEnhance	0.497 ± 0.0869
NHEK	HiCSR	0.6419 ± 0.0902
	DFHiC	**0.6547** ± 0.0892
	DiCARN	0.6509 ± 0.091
	DeepHiC	0.6351 ± 0.091
	iEnhance	0.6329 ± 0.0938
HFF-hTERT	HiCSR	0.1967 ± 0.111
	DFHiC	0.2102 ± 0.111
	DiCARN	**0.2211** ± 0.123
	DeepHiC	0.1924 ± 0.112
	iEnhance	0.19 ± 0.112

Table 8. Loop F1 Scores for Different Models Across Multiple Chromosomes

Model	Chr 4	Chr 14	Chr 16	Chr 20	Mean ± SD
iEnhance	0.2207	0.1585	0.2516	0.2442	0.2188 ± 0.0402
DeepHiC	0.2427	0.2606	0.3518	0.3715	0.3067 ± 0.0635
DFHiC	0.3079	0.3067	0.3784	0.4102	0.3508 ± 0.0519
HiCSR	0.3097	0.3021	0.3727	0.3901	0.3437 ± 0.0463
DiCARN	0.3563	0.3475	0.4145	0.4266	**0.3862 ± 0.0357**

Table 9. GenomeDISCO Average Score Comparison of DiCARN and its DNase-ased variants on 1/16 downsampled K562 Cell Line dataset

**GenomeDISCO**	**Avg**
DiCARN	0.8579
DiCARN-DNase-T	0.8599
DiCARN-DNase-S	0.8364

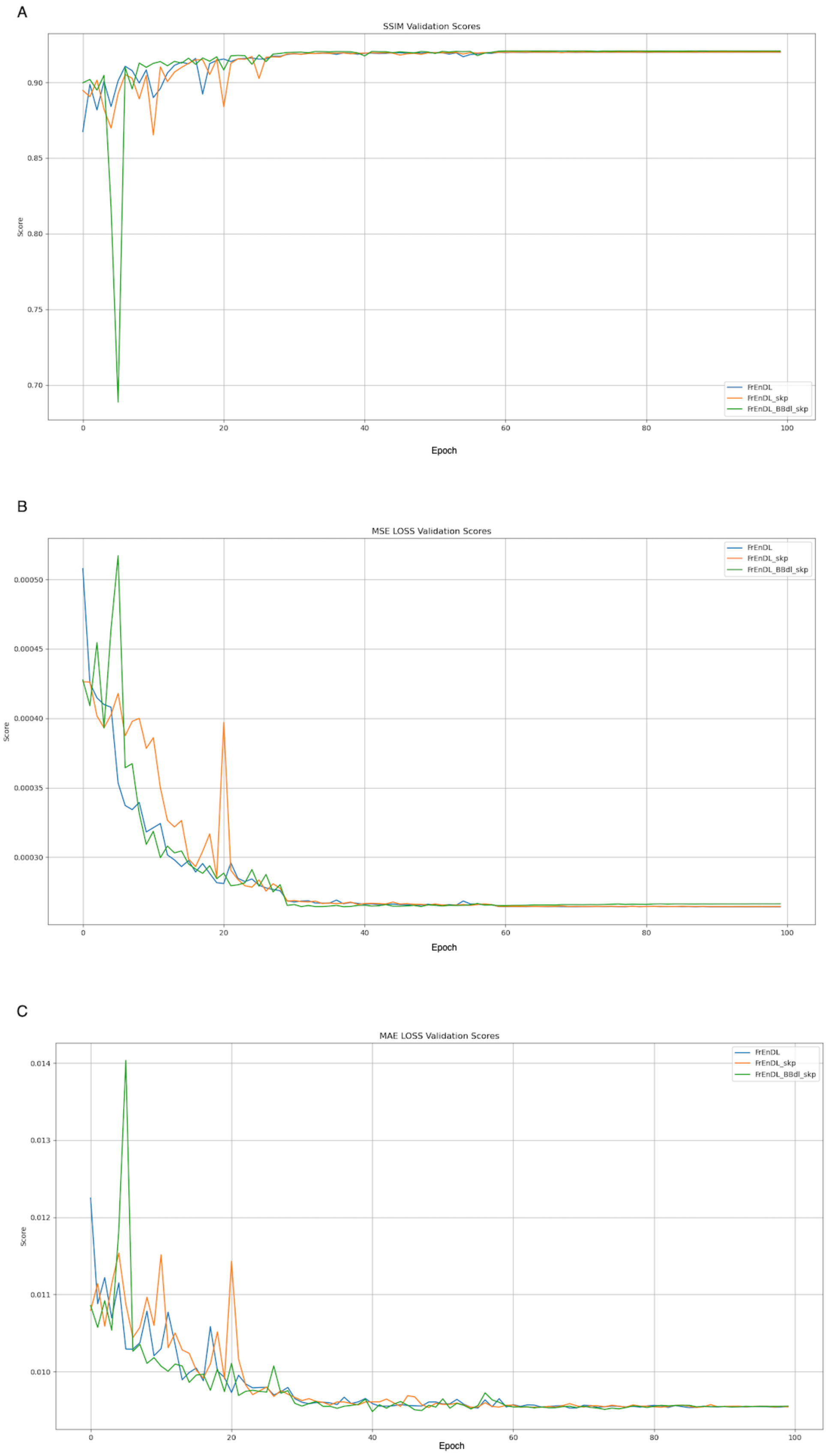

Fig. 1. Validation plots for the structural decision three skip connection implementation variations. Figure A gives the SSIM score plot, plot B shows the comparison of the MSE Loss scores, and plot C depicts the MAE loss comparison between the three variants. FrEnDL is the DiCARN architectural variant that features no skip connections; FrEnDL-skp is the version featuring a moderate amount of skip connections within each cascade layer and between cascade layers; FrEnDL-BBdl-skp is the version that highlights an additional skip connection linking the positional embeddings obtained from the first basic block layer to the final 1x1 convolutional layer at the base of the network. This experiment shows that the FrEnDL works best and was adopted as the final DiCARN version.

Table 10. Comparison of GenomeDISCO for different models and their DNase-based counterparts across various chromosomes of the 1/16 downsampled K562 cell line.

Method	Chromosome	GenomeDISCO
**DiCARN**	Chr 4	0.8491
	Chr 14	0.8571
	Chr 16	0.8568
	Chr 20	0.8688
	**Mean ± SD**	0.8579 ± 0.0081
**DiCARN-DNase**	Chr 4	0.862
	Chr 14	0.8584
	Chr 16	0.8521
	Chr 20	0.867
	**Mean ± SD**	0.8599 ± 0.0063
**HiCSR**	Chr 4	0.8390
	Chr 14	0.8050
	Chr 16	0.8206
	Chr 20	0.8501
	**Mean ± SD**	0.8287 ± 0.0199
**HiCSR-DNase**	Chr 4	0.8417
	Chr 14	0.8092
	Chr 16	0.8235
	Chr 20	0.8525
	**Mean ± SD**	0.8317 ± 0.0192
**HiCNN**	Chr 4	0.8440
	Chr 14	0.8124
	Chr 16	0.8253
	Chr 20	0.8554
	**Mean ± SD**	0.8343 ± 0.0191
**HiCNN-DNase**	Chr 4	0.8413
	Chr 14	0.8093
	Chr 16	0.8155
	Chr 20	0.8531
	**Mean ± SD**	0.8298 ± 0.0208
**DFHiC**	Chr 4	0.8440
	Chr 14	0.8124
	Chr 16	0.8253
	Chr 20	0.8554
	**Mean ± SD**	0.8343 ± 0.0191
**DFHiC-DNase**	Chr 4	0.8427
	Chr 14	0.8093
	Chr 16	0.8248
	Chr 20	0.8545
	**Mean ± SD**	0.8328 ± 0.0199
**DeepHic**	Chr 4	0.8289
	Chr 14	0.796
	Chr 16	0.81
	Chr 20	0.8409
	**Mean ± SD**	0.8180 ± 0.0199
**DeepHiC-DNase**	Chr 4	0.8217
	Chr 14	0.7873
	Chr 16	0.8061
	Chr 20	0.8355
	**Mean ± SD**	0.8127 ± 0.0207
**iEnhance**	Chr 4	0.8327
	Chr 14	0.7979
	Chr 16	0.8135
	Chr 20	0.8466
	**Mean ± SD**	0.8227 ± 0.0214
**iEnhance-DNase**	Chr 4	0.4103
	Chr 14	0.3374
	Chr 16	0.4517
	Chr 20	0.5094
	**Mean ± SD**	0.4272 ± 0.0724

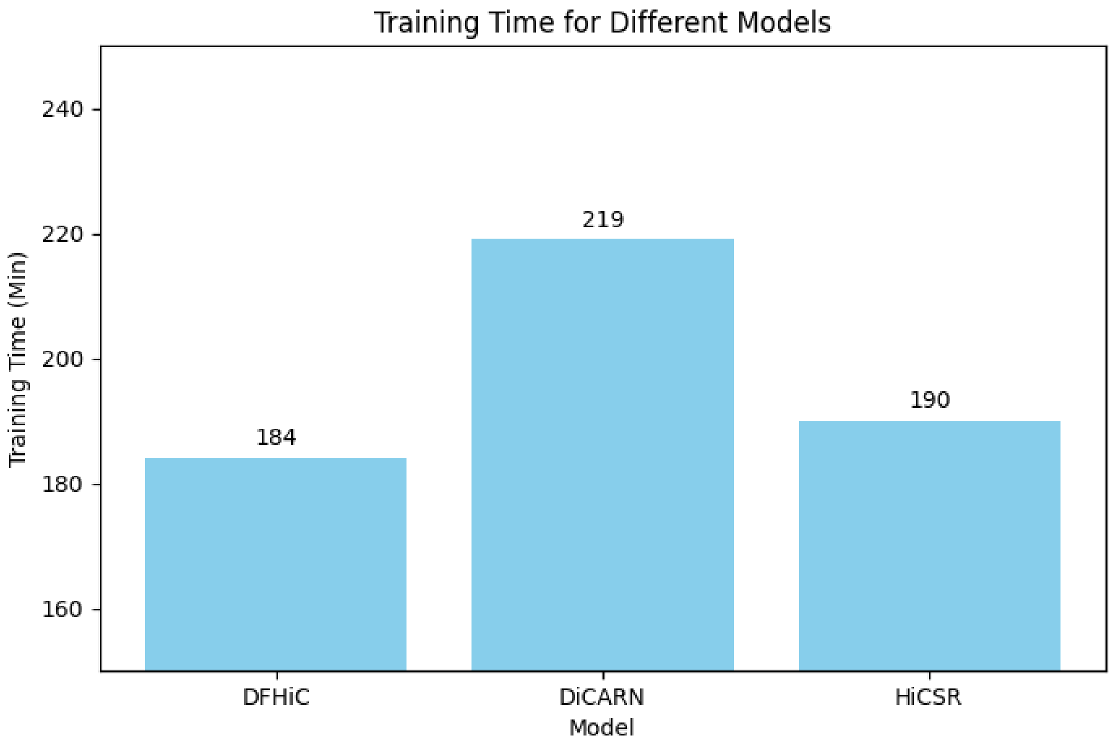

Fig. 2. Time taken to train each model per 100 epochs.

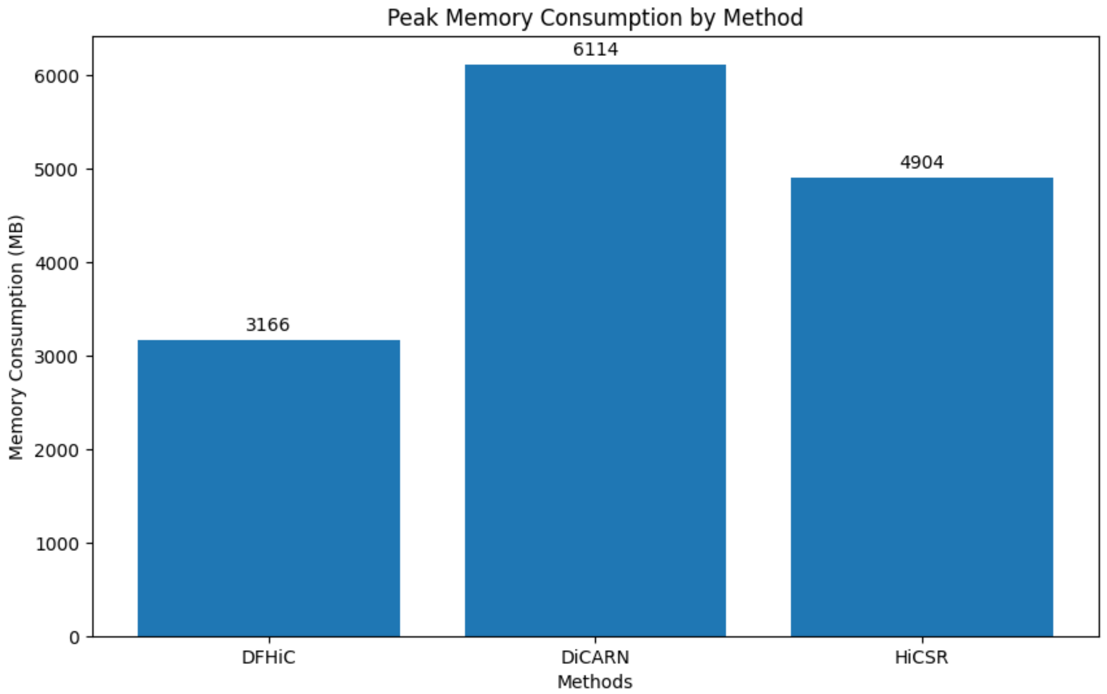

Fig. 3. Peak memory consumption by each model at training.

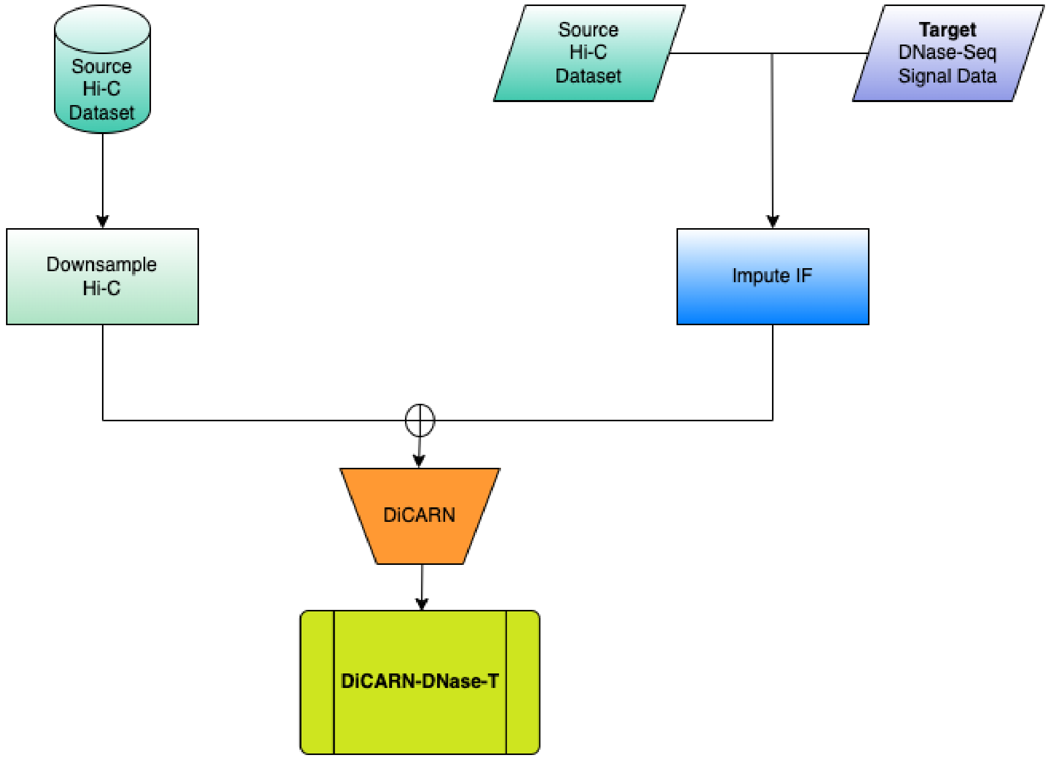

Fig. 4. The pictorial illustration of the DiCARN-DNase-T

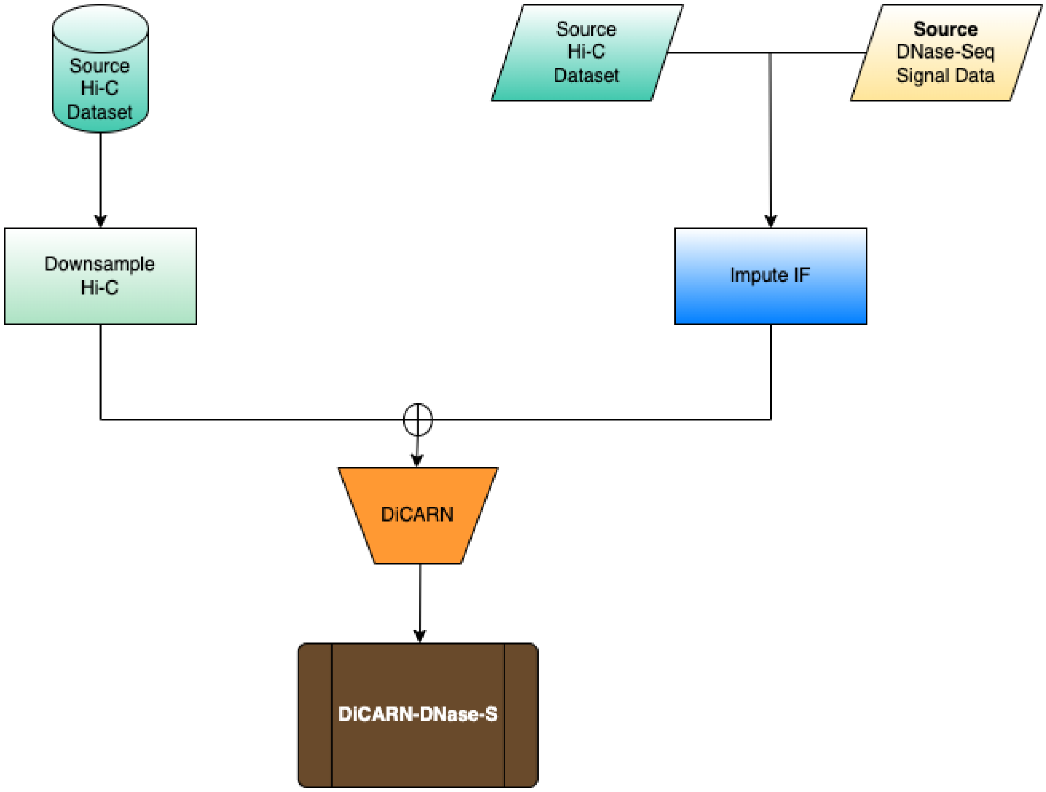

Fig. 5. The pictorial illustration of the DiCARN-DNase-S

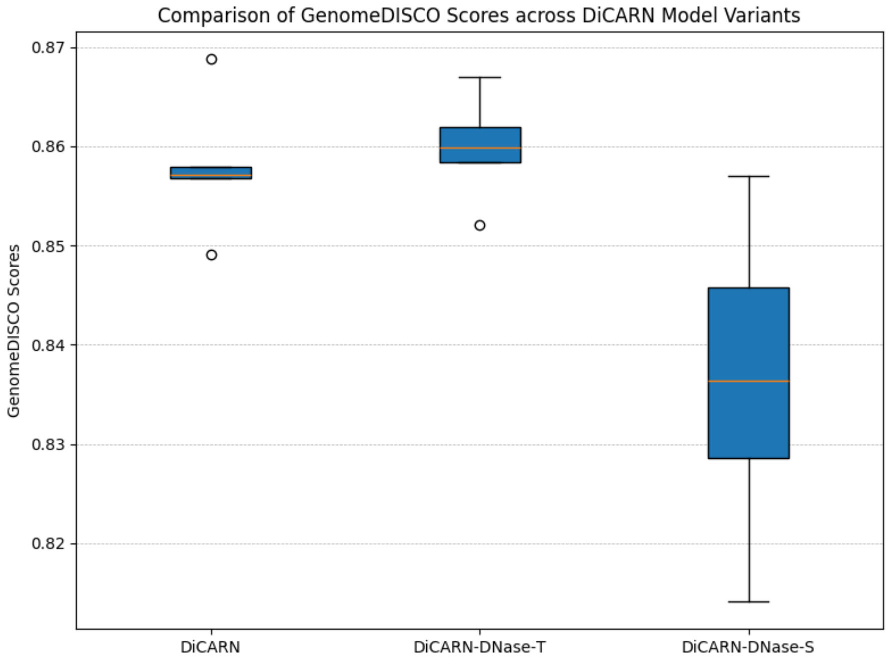

Fig. 6. The biological concordance score using GenomeDISCO for DiCARN is contrasted against the Target DNase and Source DNase variants. These results are based on the 1/16 downsampled K562 dataset.

Table 11. Comparative Performance Rankings of DiCARN and Other Models Across Cell Lines. Lower figures indicate higher ranking or better performance (i.e., 1 = best performer).

Cell	DiCARN	HiCSR	HiCARN	DFHiC	HiCNN	DeepHiC	iEnhance
K562	1	5	2	6	3	4	7
HMEC	5	1	4	3	6	2	7
NHEK	2	3	5	4	7	1	6

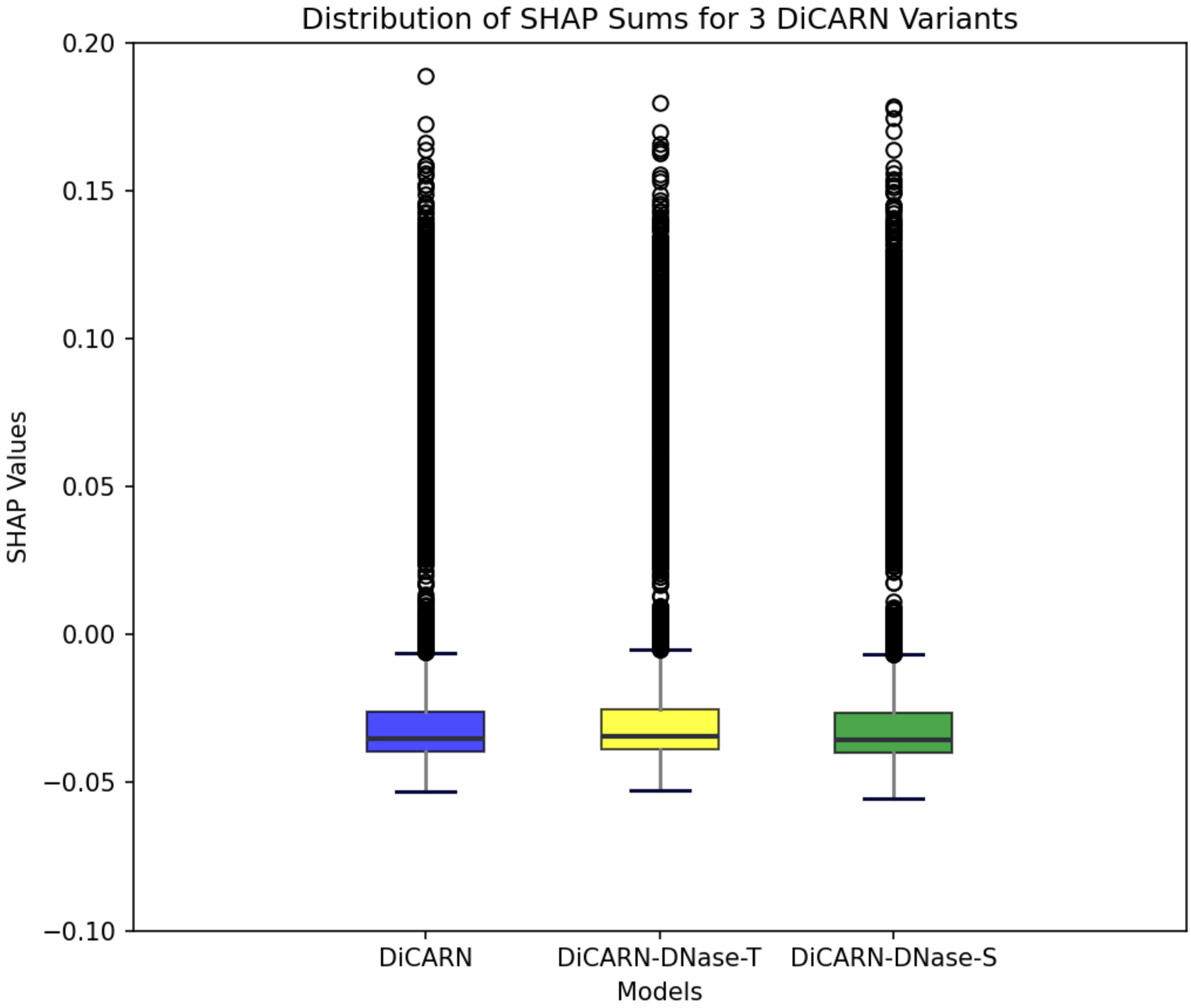

Fig. 7. SHAP distribution analysis Boxplots of the 3 DiCARN variants.

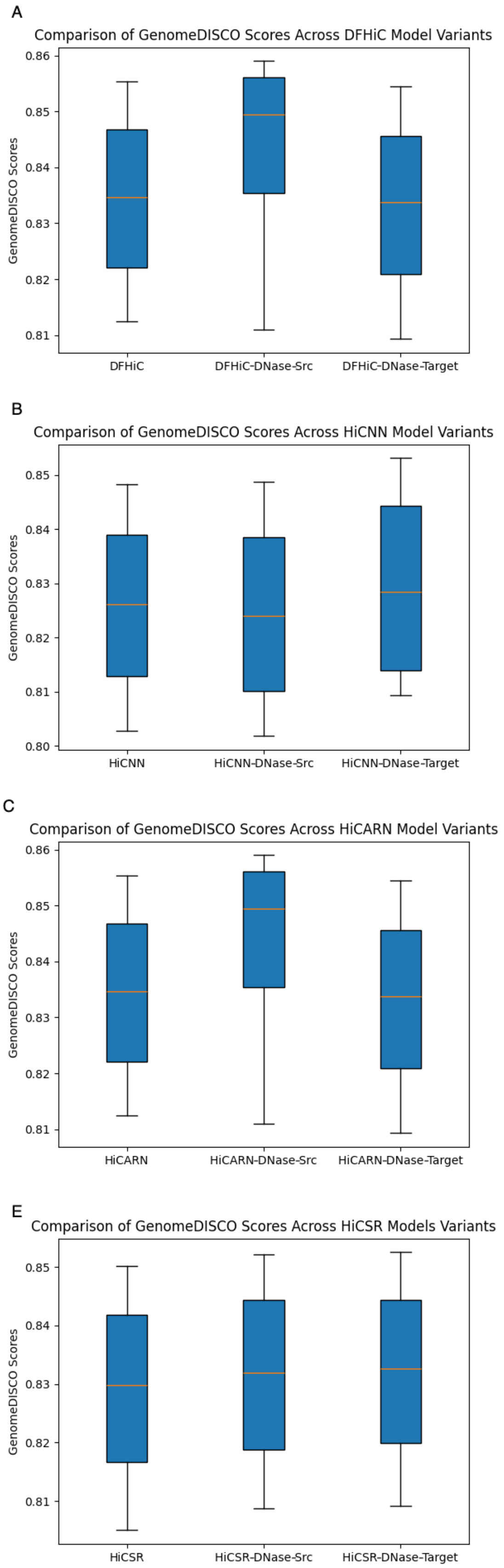

Fig. 8. The biological concordance score using GenomeDISCO for all other vanilla models is compared to their corresponding DNase-induced version. This experiment is performed using the 1/16 downsampled K562 datasets.

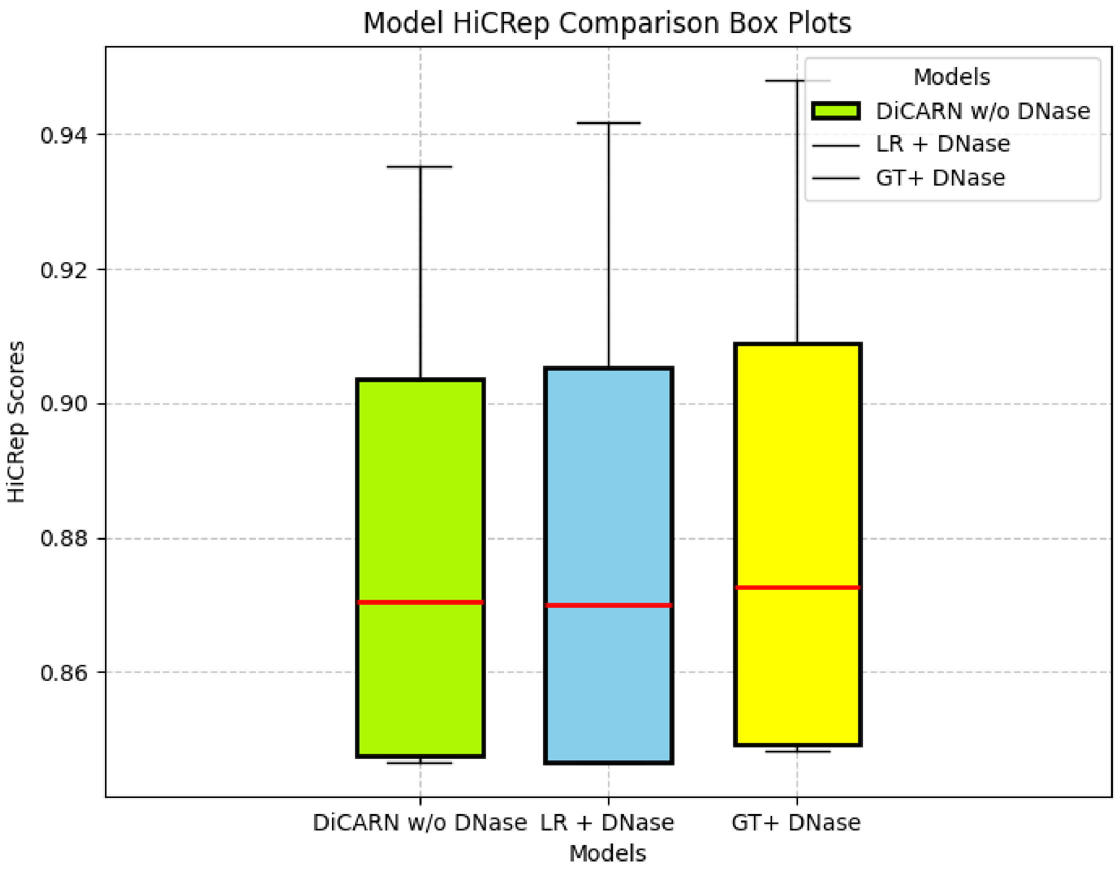

Fig. 9. HiCRep Boxplot visualization for the exposition of DNase impact

References

1. A. Vaswani, N. Shazeer, N. Parmar, J. Uszkoreit, L. Jones, A. N. Gomez, Ł. Kaiser, and I. Polosukhin. Attention is all you need. *Advances in neural information processing systems*, 30, 2017.
